# Mitochondria-sequestered A**β** renders synaptic mitochondria vulnerable in the elderly with a risk of Alzheimer disease

**DOI:** 10.1172/jci.insight.174290

**Published:** 2023-11-22

**Authors:** Kun Jia, Jing Tian, Tienju Wang, Lan Guo, Zhenyu Xuan, Russell H. Swerdlow, Heng Du

**Affiliations:** 1 Department of Pharmacology and Toxicology, University of Kansas, Lawrence, Kansas, USA.; 2 Department of Biological Sciences, Center for Systems Biology, University of Texas at Dallas, Richardson, Texas, USA.; 3 Alzheimer’s Disease Center, University of Kansas Medical Center, Kansas City, Kansas, USA.

**Keywords:** Aging, Neuroscience, Alzheimer disease, Mitochondria, Mouse models

## Abstract

Mitochondria are critical for neurophysiology, and mitochondrial dysfunction constitutes a characteristic pathology in both brain aging and Alzheimer disease (AD). Whether mitochondrial deficiency in brain aging and AD is mechanistically linked, however, remains controversial. We report a correlation between intrasynaptosomal amyloid β 42 (Aβ42) and synaptic mitochondrial bioenergetics inefficiency in both aging and amnestic mild cognitive impairment, a transitional stage between normal aging and AD. Experiments using a mouse model expressing nonmutant humanized Aβ (humanized Aβ-knockin [hAβ-KI] mice) confirmed the association of increased intramitochondrial sequestration of Aβ42 with exacerbated synaptic mitochondrial dysfunction in an aging factor- and AD risk–bearing context. Also, in comparison with global cerebral Aβ, intramitochondrial Aβ was relatively preserved from activated microglial phagocytosis in aged hAβ-KI mice. The most parsimonious interpretation of our results is that aging-related mitochondrial Aβ sequestration renders synaptic mitochondrial dysfunction in the transitional stage between normal aging and AD. Mitochondrial dysfunction in both brain aging and the prodromal stage of AD may follow a continuous transition in response to escalated intraneuronal, especially intramitochondrial Aβ, accumulation. Moreover, our findings further implicate a pivotal role of mitochondria in harboring early amyloidosis during the conversion from normal to pathological aging.

## Graphical abstract

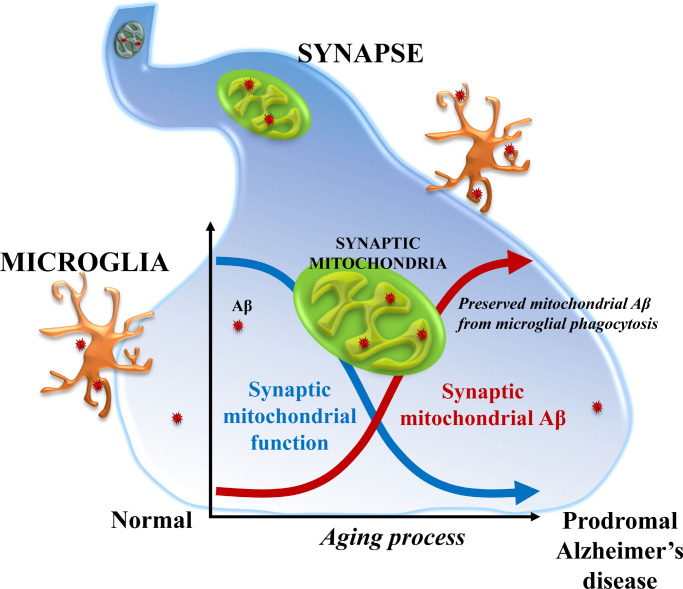

## Introduction

Late-onset Alzheimer disease (AD) that lacks disease-causing genetic risks accounts for most AD cases (1), but the precise mechanisms that divert memory disturbances from an aging trajectory to dementia are not fully understood. Although it has not been settled whether advanced aging and AD lie along the same path, mitochondrial dysfunction has been consistently identified as a common pathological event underlying brain dysmetabolism and synaptic injury in both aging and AD paradigms (2–4). Previous studies have identified that oxidative stress, energy deficiency, and calcium deregulation accompany brain aging and also act in concert as driving stressors of neuronal damages in AD (5–10). The role of mitochondria as the nexus of cellular redox balance, ATP generation, and calcium homeostasis (2, 10–12) thus underpins a possible mitochondrial link between aging and AD (2). However, whether mitochondrial dysfunction in aging and AD is mechanistically linked is complicated by the sophisticated interaction between mitochondria and AD-associated pathological molecules (13, 14). Amyloid β (Aβ) is a well-characterized mitochondrial toxin in AD-related conditions (15–20). In addition to its deleterious impacts on glucose metabolism (21–24) and cell signaling pathways (25–28) that cause defects in mitochondrial biology, Aβ also directly targets mitochondria and trespasses inside to foster severe mitochondrial dysfunction (15, 16, 19, 29–31). Notably, although brain amyloidosis is predominantly used to define AD pathology (32), neuroimaging data have shown an association of progressive Aβ accumulation in normal aging brains with longitudinal cognitive decline and AD risk in asymptomatic older adults (33–35). Taking into consideration mitochondrial sensitivity to Aβ, an interesting and yet unresolved question, is raised, therefore, of whether aging- and AD-related mitochondrial dysfunction follows a continuous transition during which aging factors and Aβ intersect to render mitochondria vulnerable to AD risk in older adults.

Amnestic mild cognitive impairment (aMCI) constitutes an intermediate stage, both symptomatically and pathologically, between aging and AD (36, 37). Older adults with aMCI do not meet the diagnostic criteria of dementia but are at a greater risk to later develop AD (38, 39). In contrast to their heterogeneity in brain pathological profiles, including cerebral Aβ deposition, tauopathy, and synaptic loss (9, 40–43), patients with aMCI demonstrate characteristic energy dysmetabolism alongside increased oxidative stress in the neocortex and hippocampus (9, 40). These metabolic deficits implicate impaired mitochondrial bioenergetics in aMCI and offer a research opportunity to examine whether Aβ is involved in mitochondrial dysfunction in this unique stage of the AD continuum.

Here, we report decreased cytochrome c oxidase–dependent (CcO-dependent) respiration of synaptic mitochondria from patients with aMCI. In addition, we have observed an association of intrasynaptosomal Aβ42, but not global cerebral Aβ, with CcO defects alongside mitochondrial Aβ accumulation in both aged cognitive norms and patients with aMCI. Further examination using a mouse model with AD risk (humanized Aβ-knockin [hAβ-KI] mice, also referred to as a mouse model of late-onset AD in refs. 44 and 45) confirmed the influence of mitochondrial Aβ42 on synaptic mitochondrial function and showed a role of synaptic mitochondria in preserving Aβ from microglia-mediated removal. These findings suggest that mitochondrial sequestration of Aβ42 may contribute to synaptic mitochondrial deficits during the transition from normal to pathological aging. Moreover, synaptic mitochondria constitute a pivotal site in harboring early amyloidosis in the elderly with AD risk.

## Results

### Increased synaptosomal Aβ42 in patients with aMCI.

**Figure 1:**
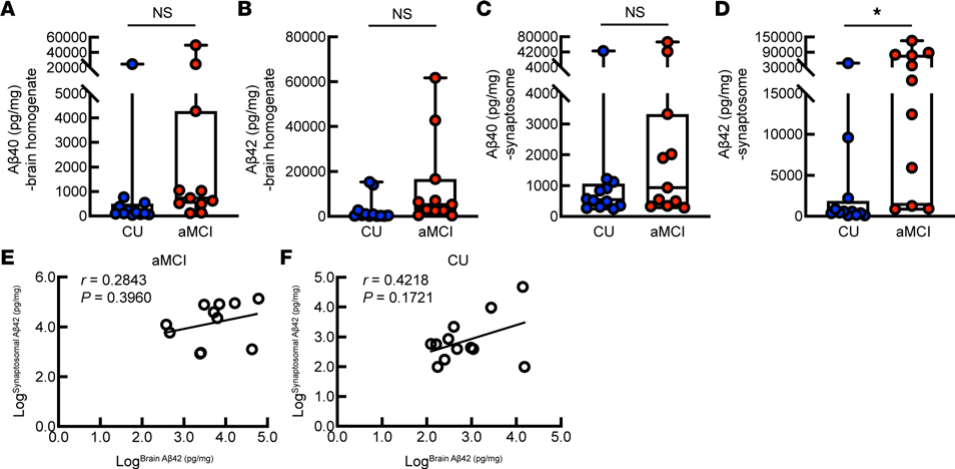
Brain and synaptosomal amyloidosis in CU individuals and patients with aMCI. (**A** and **B**) Brain Aβ40 (**A**) and Aβ42 (**B**) in CU individuals and those with aMCI measured by ELISA. Two-tailed *t* test with Welch’s correction. CU, *n* = 12; aMCI, *n* = 11. (**C** and **D**) Synaptosomal Aβ40 (**C**) and Aβ42 (**D**) in CU individuals and those with aMCI measured by ELISA. Two-tailed Mann-Whitney *U* test (**C**) and Welch’s *t* test (**D**). CU, *n* = 12; aMCI, *n* = 11. (**E** and **F**) Correlation analysis of brain and synaptosomal Aβ42 for patients with aMCI (**E**) and CU individuals (**F**). Pearson’s correlation coefficients. CU, *n* = 12; aMCI, *n* = 11. *P < 0.05.

To determine whether brain Aβ load differentiates preclinical AD from normal aging, we performed ELISAs for Aβ40 and Aβ42 in postmortem temporal lobe tissues from 11 patients with aMCI and 12 age- and sex-matched cognitively unimpaired (CU) control individuals (Supplemental Table 1; supplemental material available online with this article; https://doi.org/10.1172/jci.insight.174290DS1). The temporal pole was selected because it demonstrates early pathological changes in AD (46). Further data analysis showed no difference in either Aβ40 (Figure 1A) or Aβ42 (Figure 1B) between the 2 groups, suggesting a limited capacity of brain Aβ load in distinguishing prodromal AD from normal aging, at least, in the tested cohort. Corroborating comparable cerebral Aβ, further fluorometric assays showed no difference in β-secretase 1 (BACE1) activity between patients with aMCI and the CU control individuals (Supplemental Figure 1). Emerging evidence highlights the importance of intracellular Aβ deposition to neuronal perturbations in AD (47). To this end, we extended our observations to amyloidosis in neurons by ELISA examination of Aβ40 and Aβ42 in isolated synaptosomal fractions. The purity of synaptosomes was determined by electron microscopy (EM) (Supplemental Figure 2). In contrast to comparable levels of synaptosomal Aβ40 (Figure 1C), increased synaptosomal Aβ42 was determined in patients with aMCI (Figure 1D); however, there was no correlation between synaptosomal and cerebral Aβ42 in either patients with aMCI (Figure 1E) or CU individuals (Figure 1F). In view of previous reports of the close association of intraneuronal Aβ accumulation with neuronal injury and cognitive deficits (47–49), these findings seem to imply a link between increased intraneuronal Aβ42 deposition and the development of prodromal AD.

### Negative relationship between synaptic mitochondrial bioenergetics and synaptosomal Aβ42 in both aging individuals and those with aMCI.

Owing to their proficiency in ATP generation, synaptic mitochondria support energy-demanding synaptic activity and cognitive function (50). To determine whether increased intrasynaptosomal Aβ42 deposition affects synaptic mitochondrial bioenergetics in patients with aMCI, we performed biochemical assays for the activity of CcO, the deficiency of which has been consistently reported to be a common cause of mitochondrial crisis in both aging and AD brains (51–56).

**Figure 2:**
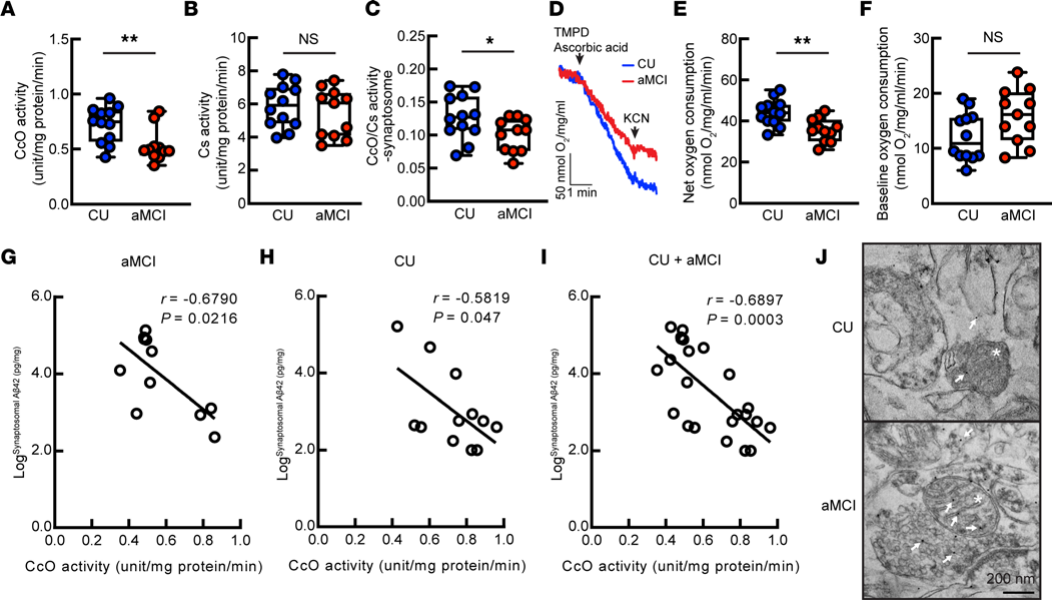
Synaptic mitochondrial bioenergetics and their correlation with synaptosomal Aβ in CU individuals and patients with aMCI. (**A**–**C**) Mitochondrial CcO (**A**) and Cs (**B**) activity and CcO to Cs ratio (**C**) in CU and aMCI brain synaptosomal fractions. Unpaired 2-tailed *t* test. CU, *n* = 12; aMCI, *n* = 11. (**D**–**F**) Mitochondrial oxygen consumption in CU and aMCI synaptosomes. (**D**) Oxygraph generated by a Clark-type electrode. (**F**) Baseline oxygen consumption was recorded by inhibiting CcO activity using KCN. (**E**) Net oxygen consumption was calculated by subtracting baseline oxygen consumption from induced oxygen consumption. Unpaired 2-tailed *t* test. CU, *n* = 12; aMCI, *n* = 11. (**G**–**I**) Correlation of synaptic mitochondrial CcO activity and synaptosome Aβ42 in patients with aMCI (**G**), CU individuals (**H**), and combined individuals of the 2 groups. (**I**) Pearson’s correlation coefficients. CU, *n* = 12; aMCI, *n* = 11. (**J**) Immunogold labeling of Aβ. Arrows indicate positively stained Aβ particles. Mitochondria are showed by asterisks. Scale bar: 200 nm. *P < 0.05, **P < 0.01. TMPD, *N*,*N*,*N′*,*N′*-tetramethyl-*p*-phenylenediamine.

As compared with their counterparts from CU control individuals, lower synaptic mitochondrial CcO activity was determined in patients with aMCI (Figure 2A). The unchanged activity of citrate synthase (Cs) (Figure 2B) and the resultant decrease in the CcO activity to Cs activity ratio (Figure 2C) further confirmed the specificity of synaptic mitochondrial CcO inefficiency in aMCI. Mitochondrial oxygen consumption is a sensitive indicator of mitochondrial bioenergetics capacity, and CcO is the key enzyme responsible for oxygen-consuming redox respiration (57). Therefore, we examined mitochondrial oxygen consumption triggered by the artificial CcO substrate *N*,*N*,*N*′,*N*′-tetramethyl-*p*-phenylenediamine in the presence of ascorbic acid as an electron donor (58). A decreased net synaptic mitochondrial oxygen consumption rate was determined in patients with aMCI (Figure 2, D and E), with comparable baseline oxygen consumption as CU individuals (Figure 2F), supporting impaired mitochondrial bioenergetics in this prodromal stage of AD.

To examine the influence of Aβ on synaptic mitochondrial function, we performed correlation analysis and found a negative association of CcO activity with synaptosomal Aβ42 not only in patients with aMCI (Figure 2G) but also in CU control individuals (Figure 2H) and the aMCI and CU combined group (Figure 2I). In contrast, no correlation was observed between CcO activity and synaptosomal Aβ40 (Supplemental Figure 3, A–C), brain Aβ42 (Supplemental Figure 3, D–F), or brain Aβ40 (Supplemental Figure 3, G–I) in any tested group. These findings implicate a deleterious impact of synaptosomal Aβ42 on synaptic mitochondrial function in both normal and pathological aging.

Mitochondria-sequestered Aβ constitutes an integral part of intraneuronal amyloidosis and directly affects mitochondrial function (15, 16, 19, 29–31). Because of the technical difficulties involved in purifying intact synaptic mitochondria from frozen brain tissues to avoid the contamination of extramitochondrial Aβ during sample preparation, we performed immunogold EM to detect synaptic mitochondrial Aβ. Echoing the relationship between synaptosomal Aβ and CcO activity (Figure 2, G–I), EM examination identified immunogold-labeled Aβ within synaptic mitochondria in both aMCI and CU brains (Figure 2J). Being aware of the potential influence of tauopathy on mitochondrial function (59), we compared tauopathy by proxy of the Braak score and found no correlation between CcO activities with the Braak scores in either patients with aMCI (Supplemental Figure 4A) or their CU counterparts (Supplemental Figure 4B). Taken together, our findings support an association of synaptosomal Aβ42 and also possibly intramitochondrial Aβ with synaptic mitochondrial decay in aging and aMCI brains, thus implying a role of intraneuronal Aβ42 in mediating continuous mitochondrial changes during the conversion from normal to pathological aging.

### Increased intramitochondrial Aβ42 accumulation in hAβ-KI mice with aging.

Our observations in human samples implicate a potential contribution of intraneuronal, especially intrasynaptic, mitochondrial Aβ to the development of synaptic mitochondrial dysfunction with aging, which may possibly potentiate the deviation of cognitive decline from a normal to a pathological aging-related track. Previous basic research on AD-related mitochondrial dysfunction predominantly used rodent models with supraphysiological expression of Aβ and/or tau pathology, thus having limited capacity to reflect cellular perturbations in late-onset AD–relevant conditions (44). Although there is, so far, no ideal model of late-onset AD, the recently developed nonmutant hAβ-KI mice expressing the human form of Aβ exhibit changes in cognition, synaptic activity, and neuroinflammation that typify cognitive aging (44, 45). Regardless of some limitations of this model in fully recapitulating late-onset AD (44), hAβ-KI mice adequately satisfy our examination of synaptic mitochondria and amyloidosis in a chronic condition of combined aging factors and physiological levels of human form of Aβ.

**Figure 3:**
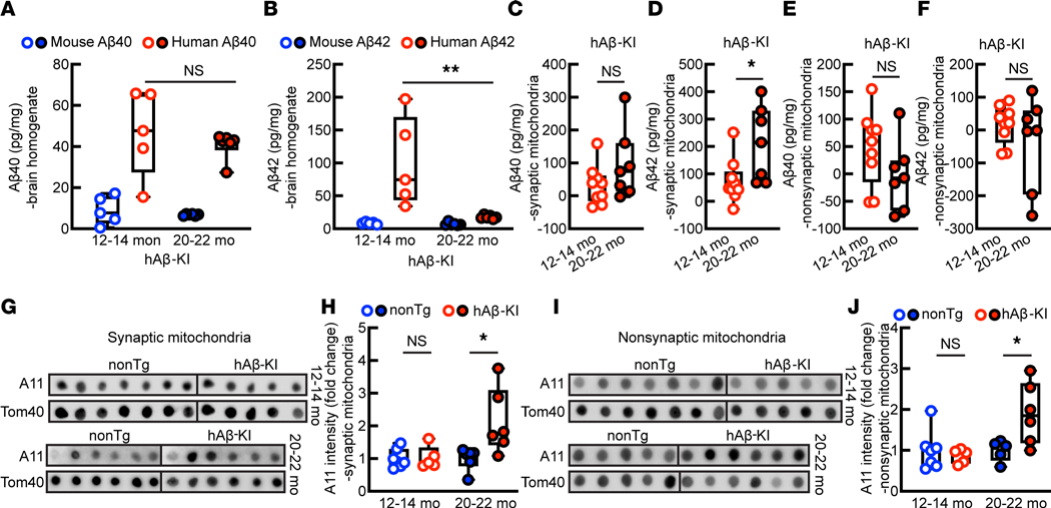
Brain and mitochondrial Aβ in hAβ-KI mice at ages 12–14 months and 20–22 months. (**A**) Aβ40 levels in brain homogenates from hAβ-KI mice. Two-tailed *t* test with Welch’s correction. Age 12–14 months, *n* = 5; 20–22 months, *n* = 6. (**B**) Aβ42 level in brain homogenates from hAβ-KI mice. Two-tailed Mann-Whitney *U* test. Age 12–14 months, *n* = 5; 20–22 months, *n* = 6. (**C** and **D**) Aβ40 (**C**) and Aβ42 (**D**) levels in synaptic mitochondria from hAβ-KI mice. Unpaired 2-tailed *t* test. Age 12–14 months, *n* = 9; 20–22 months, *n* = 7. (**E** and **F**) Aβ40 (**E**) and Aβ42 (**F**) levels in nonsynaptic mitochondria from hAβ-KI mice. Unpaired 2-tailed *t* test (**E**) and 2-tailed *t* test with Welch’s correction (**F**). Age 12–14 months, *n* = 9; 20–22 months, *n* = 7. (**G** and **H**) Aβ oligomers in synaptic mitochondria. (**G**) Representative images of A11 and Tom40 dot blotting; (**H**) Aβ oligomers labeled by A11 Ab. Age 12–14 months: unpaired 2-tailed *t* test, nonTg, *n* = 7; hAβ-KI, *n* = 5. Age 20–22 months: 2-tailed *t* test with Welch’s correction, *n* = 6 each group. (**I** and **J**) Aβ oligomers in nonsynaptic mitochondria. (**I**) Representative images of A11 and Tom40 dot blotting; (**J**) Aβ oligomers labeled by A11 Ab. Unpaired 2-tailed *t* test. Age 12–14 months: nonTg, *n* = 7; hAβ-KI, *n* = 5. Age 20–22 months: nonTg, *n* =5; hAβ-KI, *n* = 6. *P < 0.05, **P < 0.01.

To determine whether synaptic mitochondrial accumulation of Aβ increases in hAβ-KI mice during aging, we performed ELISA for Aβ in brain homogenates and mitochondrial fractions from hAβ-KI mice ages 12–14 and 20–22 months. The age- and sex-matched non–gene-manipulated (nontransgenic [nonTg]) mice expressing endogenous murine Aβ were used to control the aging factors in a human Aβ–free condition. The mouse ages used were determined on the basis of a previous report (44) and our own observations of preserved recognition memory (Supplemental Figure 5A) and synaptic density in the hippocampal CA1 region (Supplemental Figure 5B) in hAβ-KI mice at 12–14 months old and decreased cognitive performance (Supplemental Figure 5C) and hippocampal CA1 synaptic density (Supplemental Figure 5D) in these mice at 20–22 months old. As expected, ELISAs for Aβ40 and Aβ42 in brain homogenates showed the expression of human form Aβ40 (Figure 3A) and Aβ42 (Figure 3B) with nondetectable murine Aβ in hAβ-KI mice. In contrast to no age effect on brain Aβ40 (Figure 3A), hAβ-KI mice surprisingly displayed an age-dependent decrease in brain Aβ42 (Figure 3B). In a parallel study, such a brain Aβ change in hAβ-KI mice was not observed in nonTg mice (Supplemental Figure 6, A and B), suggesting that decreased brain Aβ42 is not likely to be an age-related phenotype in mice.

To determine whether such a change in brain Aβ42 is due to loss of guanidine-insoluble Aβ oligomers and/or fibrils during sample preparation, we then performed dot-blotting for Aβ oligomers in brain homogenates using, A11, a specific Ab against Aβ oligomers (60) and IHC staining with Congo red, which detects Aβ fibrils as well as oligomers (61), in brain slices. There was no difference in brain Aβ oligomers between the 2 types of mice at both tested ages determined by A11 (Supplemental Figure 7, A and B). In addition, Congo red–positive staining was also absent in the 2 groups of mice (Supplemental Figure 7C), which agrees with a previous report (44). These results seem to support that loss of brain Aβ, especially Aβ42, is a phenotypic change of hAβ-KI mice with aging.

Next, we purified synaptic and nonsynaptic mitochondria from hAβ-KI mice at ages 12–14 and 20–22 months. The purity of isolated mitochondrial fractions was determined by the abundance of voltage-dependent anion channel 1, a specific mitochondrial protein, and the absence of cytosolic proteins including β-actin and ER-specific calnexin (Supplemental Figure 8A) as well as EM examination (Supplemental Figure 8B). Synaptic mitochondria in hAβ-KI mice had a stable amount of Aβ40 during aging (Figure 3C). However, in contrast to the age-dependent reduction in brain Aβ42 (Figure 3B), synaptic mitochondrial fractions from hAβ-KI mice demonstrated increased Aβ42 in an age-dependent manner (Figure 3D). Notably, nonsynaptic mitochondria from hAβ-KI mice did not exhibit any age-related changes in Aβ40 (Figure 3E) or Aβ42 (Figure 3F), indicating that accumulation of Aβ42 predominantly develops in synaptic mitochondria. Intriguingly, increased mitochondrial accumulation of Aβ42 was also observed in old, nonTg control mice, supporting an age effect regardless of genotype (Supplemental Figure 9, A–D). In addition, as compared with their nonTg counterparts, synaptic mitochondria from hAβ-KI mice had increased Aβ oligomerization, determined by dot-blotting with A11 (Figure 3, G and H). Although nonsynaptic mitochondria from younger hAβ-KI mice did not differ from their nonTg counterparts in Aβ oligomerization (Figure 3, I and J), elevated Aβ oligomerization in nonsynaptic mitochondria from hAβ-KI mice became evident with mouse aging (Figure 3, I and J). In view of the age-related changes in synaptic density and cognitive function (Supplemental Figure 5), these results cumulatively suggest that synaptic mitochondrial accumulation of Aβ42 is in line with the development of synaptic injury and cognitive deficits with aging in hAβ-KI mice.

### Synaptic mitochondrial dysfunction in hAβ-KI mice during aging.

**Figure 4:**
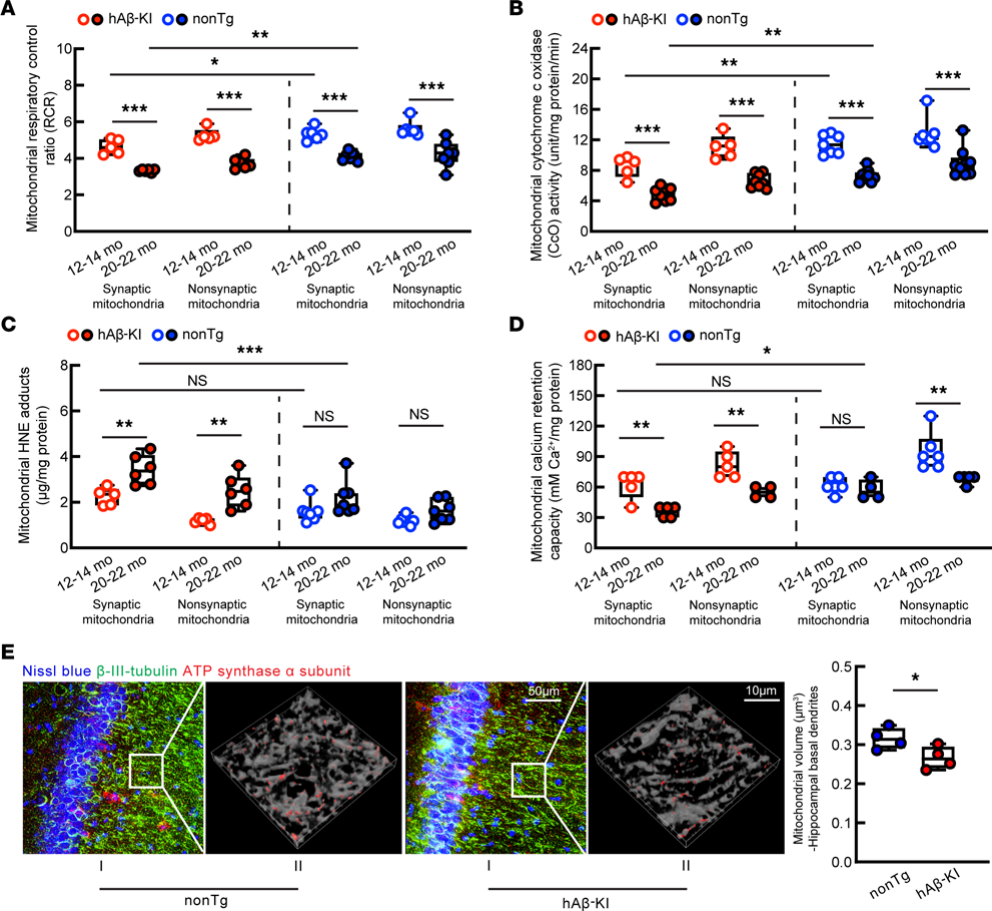
Mitochondrial function in hAβ-KI mice at ages 12–14 months and 20–22 months. (**A**) Synaptic and nonsynaptic mitochondrial RCR of hAβ-KI and age-matched nonTg mice. One-way ANOVA followed by Bonferroni’s test (nonTg, age 12–14 months, *n* = 6 and age 20–22 months, *n* =7; hAβ-KI age 12–14 months, *n* = 5 and age 20–22 months, *n* = 6). (**B**) Synaptic and nonsynaptic mitochondrial CcO activity of hAβ-KI and age-matched nonTg mice. One-way ANOVA followed by Bonferroni’s test (nonTg, age 12–14 months, *n* = 7 and age 20–22 months *n* = 9; hAβ-KI, age 12–14 months, *n* = 5 and age 20–22 months, *n* = 8). (**C**) Synaptic and nonsynaptic mitochondrial 4-hydroxynonenal (HNE) adduct levels of hAβ-KI and age-matched nonTg mice. One-way ANOVA followed by Bonferroni’s test (nonTg, *n* = 7 each group; hAβ-KI, age 12–14 months, *n* = 5 and age 20–22 months, *n* = 6). (**D**) Synaptic and nonsynaptic mitochondrial calcium retention capacity of hAβ-KI and age-matched nonTg mice. One-way ANOVA followed by Bonferroni’s test (nonTg synaptic mitochondria, age 12–14 months, *n* = 6 and age 20–22 months, *n* = 4; nonTg nonsynaptic mitochondria, age 12–14 months, *n* = 6 and age 20–22 months, *n* = 5; hAβ-KI synaptic mitochondria, *n* = 5 each group; hAβ-KI nonsynaptic mitochondria, age 12–14 months *n* = 5 and age 20–22 months, *n* = 4). (**E**) Hippocampal basal dendritic mitochondrial volume for hAβ-KI and age-matched nonTg mice at 20–22 months old. Unpaired 2-tailed *t* test. *n* = 4 mice each group: *n* = 6411, 3263, 4334, and 7212 mitochondria counted in each of the 4 nonTg mice, respectively; *n* = 3188, 1384, 1606, and 4296 mitochondria counted in each of the 4 hAβ-KI mice, respectively. Representative images of hippocampal basal dendritic mitochondria. (**E**) I. Original images. Mitochondria labeled with ATP synthase α subunit (red), basal dendrites labeled with βIII-tubulin (green). II. Mitochondria (red) inside basal dendrite (gray) in the hippocampal area. Scale bars: 50 μm and 10 μm (insets). *P < 0.05; **P < 0.01; ***P < 0.001.

To examine whether synaptic mitochondrial defects correlate with mitochondrial Aβ42, we subjected synaptic and nonsynaptic mitochondria from age- and sex-matched hAβ-KI and nonTg mice at 12–14 and 20–22 months old to various assays for the examination of mitochondrial function. Mitochondrial bioenergetics were determined by proxy of measuring glutamate- and malate-induced, NADH-linked mitochondrial respiration using a Clark electrode (19). An age-related decrease in the mitochondrial respiratory control ratio (RCR) was determined in synaptic mitochondria from hAβ-KI mice (Figure 4A). Consistent with the changes in mitochondrial respiration efficacy, an age-related reduction of CcO activity was also observed in synaptic mitochondrial fractions from hAβ-KI mice (Figure 4B). Impaired mitochondrial bioenergetics are tightly linked with oxidative stress in brain aging and AD (62). Further examination showed an age-related increase in lipid oxidation in synaptic mitochondria from hAβ-KI mice, determined by ELISA for 4-hydroxynonenal (Figure 4C).

Synaptic mitochondria play a pivotal role in the maintenance of intrasynaptic Ca^2+^ homeostasis and dampened mitochondrial Ca^2+^ handling capacity has been intensively discussed in aging- and AD-related conditions (8, 63, 64). In this regard, we examined the Ca^2+^ retention capacity of synaptic mitochondria from hAβ-KI mice and found an age-dependent decrease in Ca^2+^ retention capacity (Figure 4D), indicating compromised mitochondrial calcium handling capacity. Of note, as compared with their synaptic counterparts, the functions of nonsynaptic mitochondria were relatively preserved (Figure 4, A–D), supporting synaptic mitochondrial vulnerability in hAβ-KI mice during aging. Furthermore, synaptic and nonsynaptic mitochondrial fractions from nonTg controls also displayed an age-related effect on their functions, albeit to a lesser extent as compared with their hAβ-KI counterparts (Figure 4, A–D).

Last, dysregulated mitochondrial dynamics toward fission constitutes a phenotypic change accompanying mitochondrial functional deficits in AD neurons. To determine whether neuronal-mitochondrial morphological control is also affected in symptomatic hAβ-KI mice, hippocampal sections from hAβ-KI and nonTg mice aged 20–22 months were subjected to immunofluorescent staining using an antimitochondrial F1Fo ATP synthase α subunit to visualize mitochondria. The basal dendrites of hippocampal neurons were determined by immunostaining for βIII-tubulin as well as their unique morphology (65).

Analysis of 3D confocal images showed a genotypic reduction in the volumes of hippocampal, basal dendritic mitochondria in hAβ-KI mice (Figure 4E). The results corroborate previous findings in hAβ-KI mice (45), indicating increased neuronal mitochondrial fission in a pathological aging-related setting, which is further supported by our observations of a statistically significant decrease in the percentage of synaptic mitochondria with diameters ranging from 1.0 μm to 2.0 μm, and a slightly increased percentage of synaptic mitochondria with diameters ranging from 0.5 μm to 1.0 μm in the population of synaptic mitochondria isolated from patients with aMCI as compared with their CU counterparts (Supplemental Figure 10, A–C).

Next, to exclude the impact of tauopathy on synaptic mitochondrial function in hAβ-KI mice, we performed immunoblotting (IB) using brain extracts from aged hAβ-KI and nonTg mice and observed no discernible difference regarding the amount of total tau or phosphorylated tau at S396/S404 between the 2 groups of mice (Supplemental Figure 11). Moreover, previous studies reported that translocation of amyloid precursor protein (APP) to mitochondria may arouse mitochondrial defects (66). To this end, we continued our observations and, by IB, found comparable mitochondria-associated APP in synaptic mitochondrial fractions from the 2 genotypes of mice (Supplemental Figure 12), ruling out the impact of APP in dampening synaptic mitochondrial deficits in hAβ-KI mice. Our observations extend those of a previous report of brain mitochondrial dysfunction (45) to neuron-specific synaptic mitochondrial defects in hAβ-KI mice. Moreover, in view of synaptic mitochondrial vulnerability to accumulate amyloidosis, these findings suggest a contribution of intramitochondrial Aβ, especially Aβ42 to the development of mitochondrial dysfunction in hAβ-KI mice during aging.

### Increased Aβ production in hAβ-KI mice.

**Figure 5:**
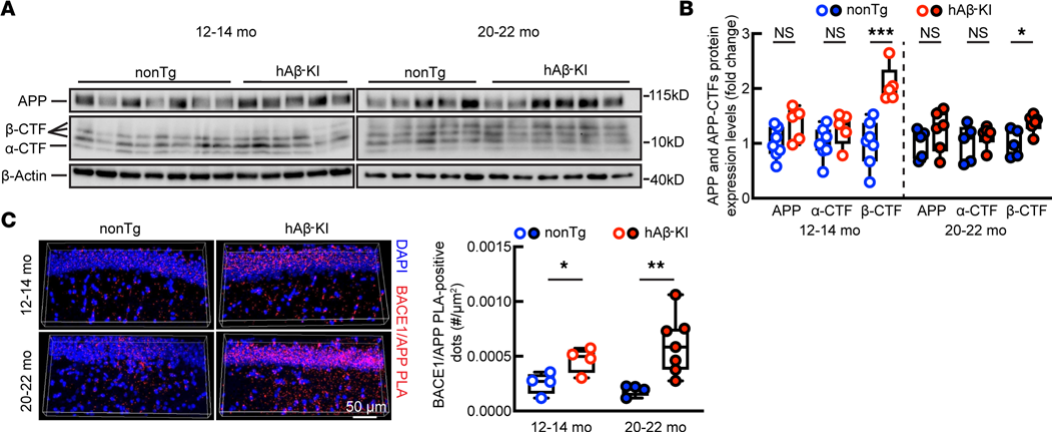
APP processing in nonTg and hAβ-KI mice at ages 12–14 months and 20–22 months. (**A** and **B**) APP and APP-CTF expression levels in nonTg and hAβ-KI brain homogenates. (**A**) Western blot images. (**B**) Analysis of APP and APP-CTF protein expression levels. The expression levels of APP-CTFs in hAβ-KI mice were normalized to the levels in nonTg mice at the same age. Unpaired 2-tailed *t* test for both ages. Age 12–14 months: nonTg, *n* = 7; hAβ-KI, *n* = 5. Age 20–22 months: nonTg, *n* = 5; hAβ-KI, *n* = 6. (**C**) APP/BACE1 Duolink PLA assay. Left: Representative images from hippocampal CA1 region. DAPI (blue); APP/BACE1 PLA dots (red). Scale bar: 50 μm. Right: Analysis of APP/BACE1 PLA dots per μm^2^. Age 12–14 months: unpaired, 2-tailed *t* test; *n* = 4 each group. Age 20–22 months: 2-tailed *t* test with Welch’s correction. nonTg, *n* = 4; hAβ-KI, *n* = 7. *P < 0.05, **P < 0.01, ***P < 0.001.

The detailed mechanisms mediating Aβ’s entry into mitochondria and the sources of intramitochondrial Aβ remain largely unresolved. A previous study reduced intraneuronal mitochondrial Aβ by blocking the receptor for advanced glycation end products (67), a cell-membrane-bound protein responsible for extracellular Aβ transport into neurons (68), suggesting that the extracellular Aβ pool is at least 1 of the major sources of intramitochondrial Aβ. This finding seems to contradict our observations of unaligned brain amyloidosis and intraneuronal Aβ in humans and hAβ-KI mice, which further prompted us to examine APP processing in hAβ-KI brains. Despite comparable APP expression, we observed increased β-carboxyl terminal fragment (β-CTF) in hAβ-KI mice at both 12–14 and 20–22 months old as compared with age- and sex-matched nonTg mice (Figure 5, A and B), indicating elevated amyloidogenic APP processing in hAβ-KI mice. However, no genotypic difference of BACE1 expression was detected by IB (Supplemental Figure 13). Of note, although no change was observed in APP (Supplemental Figure 14A) or BACE1 (Supplemental Figure 14B) via IB in brain sections of hAβ-KI mice, in situ proximity ligation assays (PLAs) for APP and BACE1 interaction showed elevated PLA-positive dots in hAβ-KI mice as compared with nonTg mice at both 12–14 and 20–22 months old (Figure 5C), suggesting increased APP interaction with BACE1 in hAβ-KI mice. The results corroborate a previous report of enhanced β-CTF interaction with BACE1 by replacing G676 in the rodent Aβ sequence with arginine, as found in human Aβ (69). Our findings thus suggest a link between augmented Aβ production and elevated intramitochondrial Aβ deposition in hAβ-KI mice, but also raise a question about the mechanisms of decreased brain amyloidosis in aged hAβ-KI mice during aging.

### Concurrence of microglial activation and less brain amyloidosis in aged hAβ-KI mice.

It is well documented that microglia play a major role in removing extracellular Aβ from the brain (70). In our in vitro experiments, ELISAs showed a culture time–dependent increase in Aβ40 and Aβ42 in the medium of primary hAβ-KI neuron cultures throughout days in vitro 3 to 12, the endpoint of our observation (Supplemental Figure 15, A and B). These results agree with our in vivo findings of increased amyloidogenic APP processing in hAβ-KI mice and further suggest preserved extracellular Aβ in a microglia-free environment.

**Figure 6:**
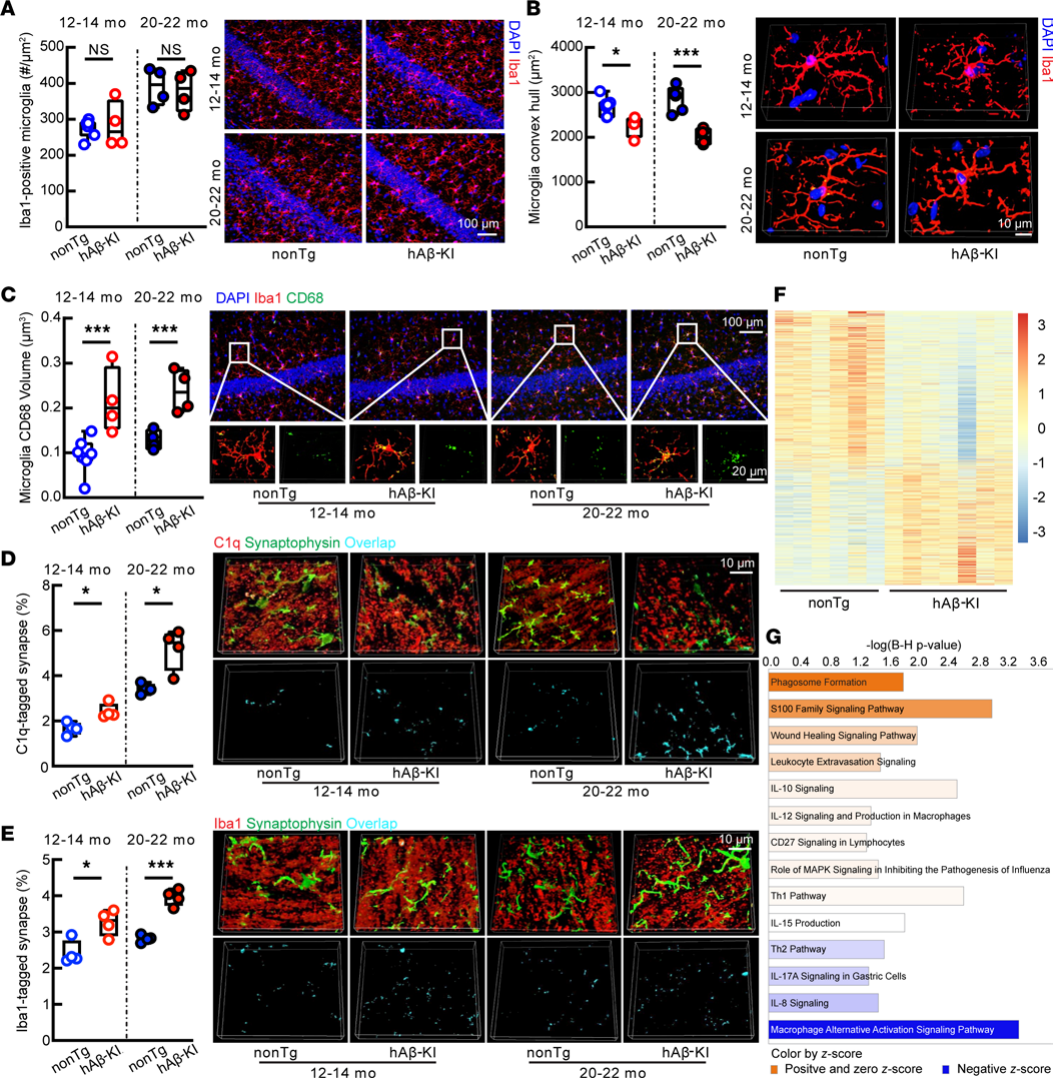
Morphology and phagocytic function of microglia in nonTg and hAβ-KI mice at ages 12–14 months and 20–22 months. (**A**) Hippocampal CA1 microglia intensity. Unpaired 2-tailed *t* test. Age 12–14 months: nonTg *n* = 7, hAβ-KI *n* = 4. Age 20–22 months: *n* = 4 each group. Right: Representative images. Scale bar: 100 μm. (**B**) Hippocampal CA1 microglia convex hull. Unpaired 2-tailed *t* test. Age 12–14 months: nonTg *n* = 7, hAβ-KI *n* = 4. Age 20–22 months: nonTg *n* = 5, hAβ-KI *n* = 4. Right: Representative images. Scale bar: 10 μm. (**C**) Hippocampal CA1 microglia CD68 volume. Unpaired 2-tailed *t* test. Age 12–14 months: nonTg *n* = 7, hAβ-KI *n* = 4. Age 20–22 months: *n* = 4 each group. Right: Representative images. Scale bar: 100 μm and 20 μm (insets). (**D**) Complement 1q–tagged (C1q-tagged) synapses in the hippocampal CA1 region. Age 12–14 months, unpaired 2-tailed *t* test; 20–22 months, 2-tailed *t* test with Welch’s correction. *n* = 4 each group. Right: Representative images. Scale bar: 10 μm. (**E**) Iba1-tagged synapses in the hippocampal CA1 region. Unpaired 2-tailed *t* test, *n* = 4 each group. Right: Representative images. Scale bar: 10 μm. (**F**) Microglial gene expression pattern of hAβ-KI compared with nonTg mice. Each column represents a sample, and each row represents a transcript. nonTg, *n* = 6; hAβ-KI, *n* = 7. (**G**) Ingenuity Pathway Analysis of differentially expressed genes in microglia (hAβ-KI versus nonTg). Orange, positive *z*-score and *z*-score of 0; blue, negative *z*-score. nonTg, *n* = 6; hAβ-KI, *n* = 7. *P < 0.05, ***P < 0.001.

If we could extrapolate the in vitro findings to an in vivo setting, we would expect activated microglial phagocytosis in aged hAβ-KI mice. To address this, we first examined microglial density in the hippocampal region of hAβ-KI and nonTg mice at ages 12–14 and 20–22 months by immunostaining for ionized calcium–binding adaptor molecule 1 (Iba1), a specific protein marker of microglia (71). Data analysis showed no genotypic or age effect on the density of Iba1-positive microglia at the tested ages (Figure 6A). Previous studies suggest that the amoeboid-like morphological changes of microglia due to process retraction indicate microglial activation and increased microglial phagocytotic capability (72). Therefore, we examined the convex hull of microglia and found decreased microglial convex hull in hAβ-KI mice as compared with nonTg controls across the tested ages (Figure 6B). Moreover, immunostaining for CD68, a sensitive indicator of microglial phagocytosis activation (73), showed increased microglial CD68 volumes in hAβ-KI mice, and the difference in microglial CD68 between hAβ-KI and nonTg mice became more prominent with aging (Figure 6C). Corroborating these changes, increased complement 1q–tagged synapses (Figure 6D) and augmented synaptophysin, the presynaptic content in microglia (Figure 6E), were seen in hAβ-KI mice. These findings of enhanced microglia-mediated synaptic pruning further provide direct evidence of activated microglial phagocytosis in hAβ-KI mice.

As a step forward, we purified microglia from aged hAβ-KI and nonTg control mice for RNA-Seq to examine microglial phagocytosis–related pathways at the transcriptomics level. In comparison with their counterparts from nonTg controls, the microglia from aged hβ-KI mice had a distinct pattern of transcriptomics profiles (Figure 6F). Further pathway analysis using Ingenuity Pathways Analysis software indicated phagosome formation highest on the list of upregulated pathways related to cellular immune response, cytokine signaling, cellular stress, and injury, as well as pathogen-influenced signaling in hAβ-KI microglia (Figure 6G). In agreement with our findings of microglial activation in symptomatic hAβ-KI mice, among the tested major proinflammatory cytokines, including *IL1*, *IL6*, and *TNFA*, that participate in AD inflammatory brain damage (74), we found remarkably increased gene expression of *TNFA* in aMCI brains (Supplemental Figure 16, A–C). In addition, *IL10*, an antiinflammatory cytokine (75), remained unchanged across aMCI and CU brains (Supplemental Figure 16D). These results indicate elicited neuroinflammation in aMCI brains and further implicate an early microglial activation accompanying pathological aging.

Together, our results indicate activated microglial phagocytosis in hAβ-KI mice, which may contribute to extracellular Aβ removal. The concurrence of increased microglial phagocytic capacity, decreased brain amyloidosis, and elevated intrasynaptic mitochondrial Aβ also implicates a role of synaptic mitochondria in protecting amyloidosis from microglia-mediated clearance in aging factor– and AD risk–bearing conditions.

## Discussion

Although aging is the greatest risk factor for AD, and mitochondrial dysfunction also constitutes a pathological characteristic of aging brains (10, 76, 77), whether mitochondria dysfunction in aging and AD is mechanistically linked remains a longstanding scientific question. In this study, we have determined a strong correlation between synaptic mitochondrial dysfunction and intrasynaptosomal Aβ42 in both CU individuals and patients with aMCI. Experiments using a mouse model expressing nonmutant humanized Aβ also showed an association of intramitochondrial accumulation of Aβ42 with synaptic mitochondrial dysfunction in an aging context. These results not only support the deleterious impact of Aβ on mitochondria in the elderly with AD risk but also indicate that Aβ’s impact on mitochondrial function also takes place in asymptomatic older adults. In line with this, it is possible that mitochondrial accumulation of Aβ42 is a pivotal factor driving a continuous deterioration of mitochondrial deficits from normal aging to dementia.

In this study, our findings of the lack of a strong relationship between synaptic mitochondrial dysfunction and global brain amyloidosis in both humans and mice accentuate the impact of mitochondrial Aβ and indicate the importance of extracellular amyloidosis to the development of neuronal mitochondrial dysfunction and cognitive impairment in pathological aging must be further questioned. Although the debate over the contribution of intra- versus extracellular Aβ deposition to AD etiopathogenesis has never reached a consensus, the clinicopathological correlation of cognitive deficits with intraneuronal neuritic plaques seems to endorse a pivotal role of intraneuronal amyloidosis in the development of AD (47, 78–82). Notably, previous studies have shown that synaptic activity promotes Aβ secretion (83, 84) but decreases intracellular Aβ42 (85), which confers to neurons resistance to Aβ-induced synaptic injury (85, 86). These findings, together with the protective effect of intraneuronal Aβ-targeted strategy in ameliorating synaptic and cognitive functions in a mouse model with familial AD–related brain amyloidosis (87), support a relevance of intraneuronal amyloidosis to synaptic failure and cognitive impairment in AD.

Mitochondria-sequestered Aβ constitutes an integral part of intraneuronal amyloidosis and has direct effects on mitochondrial function (15, 16, 19, 29–31). In this context, the qualitative and quantitative differences between mitochondrial dysfunction in aging and AD may just represent a difference in the amount of intraneuronal, especially intramitochondrial, Aβ that potentially underlies the severity of cognitive deficits. In addition, not only is there a plethora of evidence suggesting a role of Aβ in promoting mitochondrial dysfunction but also emerging evidence suggests a mitochondrial role in promoting Aβ production (88–90). Moreover, mitochondria are also a target of APP from which Aβ is cleaved (91, 92). Therefore, it could be postulated that age-related Aβ deposition in mitochondria constitutes a bona fide pathology that induces mitochondrial dysfunction and further reinforces Aβ production, culminating in the deleterious consequence of dementia. In this scenario, our results seem to support a vicious cycle of age-related mitochondrial accumulation of Aβ and mitochondrial dysfunction, which reinforce each other to promote the development of dementia from normal aging.

Another interesting issue that merits discussion is whether mitochondria are bystander victims or active players in both aging and an AD context. In this study, we saw an increase in synaptic mitochondrial Aβ42 deposition that was not aligned with decreased global brain Aβ42 in aged hAβ-KI mice. Local Aβ clearance via cellular degradation and peripheral Aβ clearance via vascular transport and cerebrospinal fluid drainage are critical mechanisms that mediate extracellular brain Aβ removal (70, 93). Although it is a limitation of the present study that we did not examine the peripheral clearance of cerebral Aβ clearance, our results suggest a contribution of activated microglial phagocytic capacity to increased cerebral Aβ elimination in aged hAβ-KI mice. In contrast, the steady increase in Aβ42 and Aβ aggregates in synaptic mitochondria accompanying aging seems to reflect the limited capability of the cerebral Aβ clearance systems to purge mitochondria-sequestered Aβ. Additionally, impaired mitochondrial removal via autophagy has been determined in aging and AD brains (94–96). Although the precise mechanisms remain unresolved, previous studies suggest a close association of defective mitophagy and mitochondrial dysfunction, including increased oxidative stress, compromised mitochondrial respiration, and impaired mitochondrial morphological control, as well as the interaction of Aβ with key molecules in mitophagy pathway in brain aging and AD paradigms (96–101). Given the correlation of mitochondrial Aβ deposition with defects of synaptic mitochondrial bioenergetics, redox balance, and dynamics determined in this study, as well as the role of mitochondrial Aβ in potentiating mitochondrial free radical production and functional deficits (15, 31, 102), it is possible that mitochondria-sequestered Aβ may also promote the dysregulation of mitophagy in older adults with AD risk, leading to impaired mitochondrial clearance through mitophagy, culminating in accumulation of damaged brain mitochondria with aging. Therefore, our findings are like to support the possibility that neuronal mitochondria undesirably cache early amyloidosis, resulting in a downward spiral of mitochondrial dysfunction and neuronal degeneration. In this case, mitochondria may play an active, rather than a passive, role in the etiopathogenesis of AD. On a related topic, the insufficient capacity to remove intraneuronal, especially intramitochondrial, Aβ may therefore constitute a therapeutic limitation of current immunotherapies that primarily target Aβ in the extracellular space.

Finally, the results of this study support a possible role of intraneuronal, especially intramitochondrial, Aβ toxicity in promoting mitochondrial dysfunction and neuronal stress during the conversion of normal to pathological aging. Although definite conclusion could only be achieved through the continuous observations of neuronal mitochondrial Aβ deposition and mitochondrial functional status in patients in the different stages of disease progression from brain aging to dementia, we cautiously propose that neuronal mitochondrial dysfunction in brain aging, and prodromal AD may follow a continuous transition that is associated with intramitochondrial Aβ deposition. Indeed, AD is not an inevitable stage of aging (76), which, to some extent, renders our hypothesis of the aging–mitochondrial dysfunction–AD sequence questionable. It should be noted that the determinant(s) of sporadic AD is still elusive. The possibility exists that a portion of the senior population have increased susceptibility to mitochondrial dysfunction during aging, which synergistically promotes the development of synaptic failure and cognitive decline with other AD-related risk factors. This hypothesis is further supported by the increasing identification of genetic factors, including mitochondrial DNA haplogroups, apolipoprotein E, several key mitochondrial proteins, and many others that may have functional consequences of mitochondrial dysfunction as well as brain and systemic dysmetabolism in patients with AD (103–109).

Despite the significance of our findings in deepening our understanding of mitochondrial biology in both aging and AD, another limitation of this study should be noted. It remains unclear whether mitochondrial accumulation of Aβ is an initiator of mitochondrial dysfunction or whether Aβ selectively accumulates in functionally impaired mitochondria during aging and the prodromal stage of AD. Furthermore, it is unknown whether mitochondria-sequestered Aβ seeds the formation of neuritic plaques with disease progression. These questions will be addressed in our future investigation. Nevertheless, the simplest interpretation of our results is that aging renders synaptic mitochondria vulnerable to Aβ accumulation and Aβ-induced functional deficits. Intraneuronal, especially intramitochondrial, Aβ42 may play a proactive role in the development of neuronal mitochondrial dysfunction during aging as well as in the transitional stage between normal aging and AD. This not only will offer us insight into the mitochondrial pathway of AD etiopathogenesis but also shed light on the development of mitochondrial Aβ–targeting approaches for the prevention of this neurodegenerative disorder.

## Methods

All the materials and methods details are available in Supplemental Methods.

### Statistics.

Data are displayed in box-and-whisker plots with all individual values shown. All statistical analysis was performed using GraphPad Prism 9 software. An unpaired, 2-tailed *t* test was used for comparisons between 2 groups when the samples were normally distributed and of equal variance. A 2-tailed *t* test with Welch’s correction was applied when both groups of data followed a normal distribution but were of unequal variance. A 2-tailed Mann-Whitney *U* test was performed when the samples were not normally distributed. One-way ANOVA followed by Bonferroni’s post hoc analysis was used for multigroup comparisons. Correlation analyses were performed using Pearson’s correlation coefficient for continuous variables or Spearman’s rank correlation coefficient for categorical variables. A *P* value of less 0.05 was considered statistically significant, and *P* values are indicated as follows in figures: ***P < 0.001, **P < 0.01, *P < 0.05.

### Study approval.

Animal studies were approved and performed under the guidelines of the University of Kansas IACUC and the NIH *Guide for the Care and Use of Laboratory Animals* (National Academies Press, 2011).

### Data availability.

Values for all data points shown in the graphs and supplemental materials are available in the Supporting Data Values file. The RNA-Seq data have been deposited in the National Center for Biotechnology Information Sequence Read Archive (accession no. PRJNA1019753).

## Author contributions

KJ, JT, HD, TW, and LG carried out experiments and collected the data. KJ, JT, ZX, and HD performed the statistical analyses. LG, RHS, and HD contributed to the design of experiments and critically read the manuscript. HD conceived the project, supervised the experiments, and wrote the manuscript.

## Supplementary Material

Supplemental data

Supporting data values

## Version 1 11/22/2023

Electronic publication
