# Supplementary material for: Mitochondria-sequestered Aβ renders synaptic mitochondria vulnerable in the elderly with a risk of Alzheimer disease
Source: JCI Insight. 2023 Nov 22;8(22):e174290. doi: 10.1172/jci.insight.174290 (PMC10721326; doi:10.1172/jci.insight.174290)
Supplement: Supplemental data [file jciinsight-8-174290-s079.pdf]

Supplemental Methods:

Reagents

D-Mannitol (MP Biomedicals, 102248), D-Sucrose (Fisher Scientific, BP220-212), Potassium Phosphate Dibasic (K_2HPO_4, Fisher Scientific, P288-500), HEPES (Fisher Scientific, BP310-100), Potassium Cyanide (KCN, Sigma-Aldrich, BP310-100), L-Ascorbic acid (Sigma-Aldrich, BP310-100), Cytochrome C from equine heart (Sigma Aldrich, C7752), Dithiothreitol (DTT, Fisher Scientific, BP172), N,N,N',N'-tetramethyl-p-phenylenediamine (TMPD, Sigma Aldrich, T3134-5G), Congo Red (Sigma Aldrich, D1306), Potassium Chloride (KCl, Fisher Scientific, P217-3), Calcium Chloride Anhydrous ($CaCl_2$, Sigma Aldrich, C1016), Nonidet P40 Substitute (NP-40, Fluka BioChemika, 74385), Tris Base (Fisher Scientific, BP152-10), Sodium Chloride (NaCl, Fisher Scientific, S271-10), Tween 20 (Thermo Scientific, J20605-AP), Paraformaldehyde (PFA, Sigma Aldrich, P6148), Triton X-100 (Sigma Aldrich, T-9284), Guanidine Hydrochloride (Sigma Aldrich, G4505), Hydrochloric Acid (HCl, Fisher Scientific, A144-500), Adenosine 5'-Diphosphate Monopotassium Salt Dihydrate (ADP, Sigma Aldrich, A5285), Sodium Hydroxide (NaOH, Fisher Scientific, S318-500), Urea (Fisher Scientific. BP169-10), Glycerol (EM Science, 56-81-5), Sodium Dodecyl Sulfate (SDS, Fisher Scientific, BP166-100), Glutaraldehyde (Electron Microscopy Sciences, 16216), Sodium Cacodylate Trihydrate (Electron Microscopy Sciences, 12300), Osmium Tetroxide (Electron Microscopy Sciences, 19112), Lead Citrate (LC, Electron Microscopy Sciences, 17800), Uranyl Acetate (UA; Electron Microscopy Sciences, 22400), Ethanol 200-Proof (Decon Laboratories, 2701), Embed 812 (Electron Microscopy Sciences, 13600), Dodecenyl Succinic Anhydride (Electron Microscopy Sciences, 13710), NADIC Methyl Anhydride (Electron Microscopy Sciences, 19000), DMP-30 (Electron Microscopy Sciences, 13600), Sodium Metaperiodate (Sigma Aldrich, 1878), Periodic Acid 1% Aqueous Solution

(Electron Microscopy Sciences, 19324), Durcupan ACM Epoxy (Electron Microscopy Sciences, 14040), Normal Goat Serum (Jackson ImmunoResearch Inc, 005-000-121).

Human

Frozen postmortem tissues from the temporal lobe were requested from the University of Texas (UT) Southwestern Medical Center ADC Neuropathology Core supported by an ADC grant (AG12300) under a protocol approved by The UT Southwestern Medical Center as well as the Brain and Body Donation Program (BBDP) at Banner Sun Health Research Institute. Patients' information was collected from all subjects by the institutions and the study adhered to the Declaration of Helsinki principles.

Mice

Animal studies were approved and performed following the guidelines of the University of Kansas Institutional Animal Care and Use Committee (IACUC) and National Institutes of Health (NIH). B6N(Cg)-App$^{tm1.1Aduci}$/J (hAβ KI) and nontransgenic (nonTg) C57BL/6N control mice were purchased from Jackson Laboratory (strain # 032013 and 005304, respectively) and bred in the KU animal care unit (ACU). Genotypes of mice were confirmed by PCR.

Novel object recognition test

Mouse novel object recognition behavioral test was performed as previously described(1). Mice were exposed to the empty testing apparatus for 5 consecutive days, 10 minutes each day during habituation. On the training day, mice were allowed to freely explore two identical objects for 10 minutes. Following a 24-hour interval, the mice underwent a 5-minute testing session where one of the objects was replaced with a novel object of a different shape. Object-exploring information was tracked and analyzed using ANY-maze software.

Primary neuron culture

Mouse primary cortical neuron culture was performed as previously described (2). Mouse cortices were dissected from Day 0 pups and kept in cold Dulbecco's modification of Eagle's medium (Corning, 10-013-CV) and dissociated in 0.08% trypsin (Gibco, 25200072) diluted in Hanks' balanced salt solution (Corning, 21-021-CV) at 37 ℃ for 15 minutes. FBS was added to inactivate trypsin and the tissue was then gently triturated in serological pipets to create a single-cell suspension. After centrifugating at 300 ×g for 5 minutes at 4 ℃, cells were resuspended in neuron culture medium containing 2% B-27 supplement (Gibco, 17504044), 2 mM GlutaMax supplement (Gibco, 35050079) in Neurobasal A medium (Gibco, 10888022). Viable cells are counted using a hemocytometer after staining with 0.4% trypan blue (Gibco, 15250061) and plated onto poly-D-lysine (P7405, Sigma-Aldrich) coated plates with proper density.

Synaptosome and mitochondria preparation

Synaptosomes, synaptic and nonsynaptic mitochondria were isolated using discontinuous Percoll (GE Healthcare, 17-0891-01) gradient centrifugation(3). All procedures were performed on ice. In brief, frozen human brain tissues or mouse brain samples were homogenized in mitochondria isolation buffer (IB buffer, 225 mM mannitol, 75 mM sucrose, 2 mM K_2PHO_4, and 5 mM HEPES; pH 7.3). After centrifuging at 1,300 ×g for 5 minutes at 4 ℃, the resulting supernatants were carefully layered onto Percoll solutions at concentrations of 15%, 23%, and 40% (v/v). Subsequent centrifugation at 34,000 ×g for 13 minutes at 4 ℃ resulted in the formation of three distinct layers. Synaptosomes were located between the 15% and 23% Percoll solutions while nonsynaptic mitochondria formed a layer between the 23% and 40% Percoll solutions. Synaptosomes and nonsynaptic mitochondria were carefully removed from the desired layers and subjected to two washes in IB buffer at 8,000 ×g for 10 minutes at 4 ℃ and pelleted for subsequent experiments. To extract synaptic mitochondria from purified synaptosomes, the synaptosome layer was

resuspended in IB buffer containing 0.02% digitonin followed by another Percoll density gradient centrifugation. Synaptic mitochondria were isolated from the layer between 23% and 40% Percoll solutions and pelleted after two washes in IB buffer.

Electron microscopy and immunogold labeling of Aβ

Transmission electron microscopy sample preparation

Purified synaptic and non-synaptic mitochondria from mouse brains, as well as synaptosomes from frozen human brain tissues, were suspended and fixed in modified Karnovsky's fixative (2% formaldehyde, 2.5% glutaraldehyde) diluted in 0.2 M cacodylate buffer/0.01 M sucrose (Su)/0.2 mM calcium chloride ($CaCl_2$). After 1 hour fixation, samples were centrifuged at 2 rpm for 5 minutes. For transmission electron microscopy (TEM) preparation, samples were rinsed in 0.2 M cacodylate buffer/Su/$CaCl_2$ and stained with 2% osmium tetroxide for 4 hours. Then, samples were dehydrated in ethanol, followed by Embed 812 resin infiltration. Ultra-thin sections of the sample blocks were cut at 45 nm using a Leica ultramicrotome (Leica Microsystems) and an ultra 45° diamond knife (DiATOME, MF2658).

TEM post-embedding immunogold labeling sample preparation

Human brain tissues were cut into ~1 mm^3 cubes and fixed in modified Karnovsky's fixative, stained with 2% osmium tetroxide, and dehydrated in ethanol. Samples were embedded using Durcupan ACM epoxy. Ultra-thin sections of the sample blocks were cut at 45-70 nm using a Leica UC6 ultramicrotome. Grids were blocked with 3% normal goat serum 10 minutes and incubated with β-Amyloid (D54D2) XP rabbit primary antibody (1:100, Cell Signaling Technology, 8243) overnight at 4 °C. Next day, grids were rinsed and incubated with 12 nm Colloidal Gold AffiniPure goat anti-rabbit secondary antibody (1:40, Jackson ImmunoResearch Inc, 111-205-144) for 2 hours at room temperature. The grids were then counterstained with 4%

uranyl acetate and placed on filter paper, sections side up until dry.

TEM imaging

All images were acquired at 200 kV, the condenser aperture at 1, and the objective aperture at 3 on a Hitachi H-8100 Transmission Electron Microscope (Hitachi High-Tech) with a lanthanum hexaboride (LaB6) electron gun. An AMT BioSpirit 16 mega-pixels CCD camera (Woburn, 4896 × 3264 pixels) was used with the following parameters: 4000-6000 ms exposure × 1 std. frames, gain at 4, bin at 1, gamma at 1, no sharpening, and normal contrast. All EM sample preparation, processing, and imaging was performed in the Microscopy and Analytical Imaging Resource Core Laboratory at The University of Kansas.

Aβ ELISA assay

Human or mouse Aβ40 and Aβ42 content was determined using commercial ELISA kits (Invitrogen, KMB3441 and KMB3481 for mouse Aβ40 and Aβ42, respectively; KHB3441 and KHB3481 for human Aβ40 and Aβ42, respectively). Neuron culture medium was directly diluted with the provided standard dilution buffer. Cortices, synaptosomes, and mitochondrial samples were homogenized in 5 M Guanidine HCl/ 50 mM Tris HCl and incubated overnight at room temperature. After centrifugation at 12,000 ×g for 10 minutes at 4 °C, the supernatant was diluted with the standard dilution buffer and subjected to ELISA assay following the manufacturer's instructions. All ELISA results were normalized with protein concentration.

Oxygen consumption and mitochondrial respiratory control ratio

Oxygen consumption and mitochondria respiratory control ratio were measured using a Clark type electrode (Hansatech) (4, 5). Purified synaptic and nonsynaptic mitochondria were first energized with 5 mM glutamate and 5 mM malate in IB buffer. Oxygen consumption associated with ADP utilization (stage III) was triggered by adding 200 μM ADP. Oxygen consumption due to proton

leakage from the mitochondrial inner membrane after ADP exhaustion was defined as stage IV oxygen consumption. Mitochondrial RCR was calculated as the ratio of stage III/stage IV oxygen consumption.

To record cytochrome c oxidase (CcO)-mediated oxygen consumption (induced oxygen consumption) of isolated human synaptosomes, 5 mM ascorbic acid and 5 mM TMPD were added to synaptosome suspensions. CcO-independent oxygen respiration (baseline oxygen consumption) was determined after the addition of CcO-specific inhibitor KCN.

Mitochondrial cytochrome c oxidase (CcO) activity

CcO activity was evaluated through its ability to re-oxidate ferrocytochrome c (6). Ferrocytochrome c was prepared by adding 0.05 mM DTT to cytochrome c. Ferrocytochrome c was added to synaptosome or mitochondria suspensions and then proceeded to 550 nm absorbance measurement on a Biotek NEO2 microplate reader. Mitochondrial CcO activity was calculated based on the changes in absorbance at 550 nm.

Mitochondrial calcium retention capacity (CRC)

CRC was quantified by a cell-impermeant fluorescent Ca^{2+} indicator Calcium Green-5N (Invitrogen, C3737). In brief, 10 μg of mitochondria was suspended in assay buffer containing 150 mM KCl, 5 mM HEPES, 2 mM K_2HPO4, pH 7.2 and 1 μM Calcium Green-5N. Calcium Green-5N fluorescent signal was recorded by a microplate reader with Ex/Em at 506/532 nm. After a baseline signal was recorded for 30 seconds, mitochondrial calcium uptake was triggered by adding 100 μM $CaCl_2$ solution at 2-minute intervals. The addition of $CaCl_2$ triggered a spike in signal which then returned to the baseline. The maximum amount of calcium sequestered by mitochondria was calculated as the difference between baseline and Calcium Green-5N signal.

Mitochondrial HNE measurement

Mitochondrial HNE adducts were determined using a OxiSelect HNE Adduct Competitive ELISA Kit (Cell Biolabs, Inc, STA-838) following the manufacturer's instructions.

Dot blot immunoassay

Aβ oligomer levels were detected by dot blot (7). Briefly, brain tissues and mitochondria were homogenized in PBS containing 1% NP-40. After centrifuging at 12,000 ×g for 10 minutes, 2 µl of supernatant were loaded to a nitrocellulose membrane and dried for 1 hour at room temperature. The membrane was then blocked with 5% non-fat milk in TBS-T (10 mM Tris, 0.15 M NaCl, 0.05 % Tween 20, pH 7.4) for 1 hour at room temperature and probed with rabbit anti-oligomer A11 polyclonal antibody (1:1000, Invitrogen, AHB0052) overnight at 4 ℃. After three washes with TBS-T, the membrane was incubated with HRP-conjugated goat anti-rabbit IgG (H+L) secondary antibody (1:2000, Invitrogen, 31460) for 1 hour at room temperature. Tom40 was used as the loading control for mitochondria samples. β-III-tubulin was used as the loading control for tissue homogenate. The blots were developed using the SuperSignal West Atto Ultimate Sensitivity Substrate (Thermo Scientific, A38556) and imaged with a ChemiDoc XRS+ Gel Imaging System (Bio-Rad).

BACE1 activity

BACE1 activity was measured using a commercially available kit (Abcam, ab282921) following manufacturer's instructions.

Immunocytochemistry

Briefly, mouse brains were dissected and fixed in 4% paraformaldehyde (PFA) in PBS. Brain slices at 40 µm were prepared using a Leica CM3050 S cryostat. Brain slices were blocked with blocking buffer (5% GOAT or donkey serum, 0.3% Triton-X 100 in PBS) for 1 hour in room temperature followed by overnight room temperature incubation with the following primary

antibodies in combination as needed: goat anti-Iba1 antibody (1:400, Abcam, ab5076), rat anti-CD68 antibody (1:100, BIO-RAD, MCA1957), mouse anti-C1q antibody (1:100, Abcam, ab71940), rabbit anti-synaptophysin antibody (1:400, Cell Signaling Technology, 5461S), mouse anti-PSD95 antibody (1:400, Cell Signaling Technology, 36233S), guinea pig anti-vGlut1 antibody (1:400, Synaptic Systems, 135304), rabbit recombinant anti-BACE1 antibody (1:1000, Abcam, ab183612), mouse anti-APP C-terminal fragment antibody (1:100, Biolegend, 802803), rabbit anti-ATP synthase α subunit (1:500, Abcam, ab176569), mouse anti-β-III-tubulin (1:500, Proteintech, 66375-1). After 4 times 5-minute washes with PBS, brain slices were incubated with Alex Fluor secondary antibodies conjugated with fluorophores (1:400, Alexa Fluor 488/594/647, Thermo Fisher) at room temperature for 1 hour. Cell nuclei were labeled with DAPI (Invitrogen, D1306). Neuronal soma were labeled with NeuroTrace™ 435/455 Blue Fluorescent Nissl Stain (Invitrogen, N21479). Images were captured with a Nikon Ti2 confocal microscope.

Images were processed and analyzed using Nikon NLS element software. Hippocampal CA1 APP and BACE1 expression were quantified by measuring mean fluorescent intensity. To determine synaptic density, images underwent 3D reconstruction and synaptic density was calculated as the number of vGlut1/PSD95 (pre- and post-synaptic markers, respectively) overlapping dots per um^3. Microglia density and convex hull analysis were performed as previously described (8). Microglia CD68 volume was measured as the CD68 volume in CD68/Iba-1 intersections in 3D-reconstructed images. C1q- or Iba1-tagged synapses were determined as the percentage of C1q/synaptophysin intersection or Iba1/synaptophysin intersection. Hippocampal basal dendritic mitochondria were characterized by overlapping basal dendritic marker β-III-tubulin and mitochondrial marker ATP synthase α subunit and the volume of overlapped mitochondria were measured.

Congo red staining

Parenchymal amyloid plaques were stained as previous described with Congo red (9). Brain slices were dried overnight on a microscope slide and re-hydrated in H_2O for 30 seconds. After being immersed in alkaline sodium chloride solution (saturated NaCl in 80% ethanol with 10 mM NaOH), the slices were incubated in 0.2% Congo red diluted in alkaline sodium chloride solution for 30 minutes. Afterwards, the slices were quickly dehydrated with ethanol followed by clearing in xylene for 5 minutes, 3 times. Images were collected with a Nikon Ti2 confocal microscope in brightfield mode.

Duolink In Situ proximity ligation assay (PLA)

BACE1 and APP interaction was examined using Duolink proximity ligation assay (PLA, Sigma-Aldrich, DUO92008) following the manufacture instruction. Briefly, 4% PFA-fixed mouse brain slices were blocked with the provided blocking buffer for 1 hour and then incubated with the following primary antibodies and PLA probes: rabbit recombinant anti-BACE1 antibody (1:100, Abcam, ab183612), mouse anti-APP C-terminal fragment antibody (1:100, Biolegend, 802803), Duolink In Situ PLA rabbit and mouse probes (Sigma-Aldrich, DUO92002, DUO92004, respectively). Brain slices were imaged and analyzed using a Nikon Ti2 confocal microscope.

Western blotting

Western blot samples were prepared by homogenizing brain tissues and mitochondria in urea buffer (8 M urea, 10% glycerol, 1% SDS, 5 mM DTT, and Tris-HCl, pH 6.8). Proteins were separated in either 10% Bis-Tris Gels (Invitrogen, NW00105BOX) or 10%-20% Tris-Glycine Gels (Novex, XP10205BOX) and then transferred to PVDF membranes (Bio-Rad, 1620177). Membranes were blocked with 5% non-fat milk at room temperature for 1 hour and incubated with the following primary antibodies overnight at 4 ℃: mouse anti-APP C-terminal fragment antibody (1:2000, Biolegend, 802803), rabbit anti-VDAC antibody (1:1000, Cell Signaling Technology,

4661S), rabbit anti-Calnexin antibody (1:1000, Cell Signaling Technology, 2679S), mouse anti-phospho-Tau antibody (1:1000, Cell Signaling Technology, 9632S), mouse anti-Tau (Tau46) antibody (1:1000, Cell Signaling Technology, 4019S), mouse anti-beta Tubulin antibody (1:20,000, Proteintech, 66240-1-Ig), rabbit recombinant anti-BACE1 antibody (1:1000, Abcam, ab183612), rabbit anti-TOMM40 antibody (1:3000, Proteintech, 18409-1-AP), mouse anti-beta Actin antibody (1:5000, Santa Cruz Biotechnology, sc-47778). After probing with goat anti-mouse IgG HRP conjugated or goat anti-rabbit IgG HRP conjugated secondary antibodies (1: 2,000–8,000, Thermo Fisher Scientific, 31430 and 31460), blots were developed using SuperSignal West Atto Ultimate Sensitivity Substrate (Thermo Scientific, A38556). Imaged were captured and analyzed with a ChemiDoc XRS+ Gel Imaging System (Bio-Rad).

Microglia isolation and RNA sequencing

Mice at ages of 20-22 months old were used for microglia isolation. Brain cells were separated using magnetic column-based cell separation. Each adult mouse brain was dissociated using papain (Worthington, LS003120) for 50 minutes on a shaker at 37 °C. Myelin and other cell debris was removed using Debris Removal Solution (Miltenyi Biotec, 130-109-398) and erythrocytes were removed using Red Blood Cell Lysis Solution (10x) (Miltenyi Biotec, 130-094-183). Microglia were isolated from the lysate using CD11b MicroBeads (Miltenyi Biotec, 130-093-634), respectively. Cells were pelleted down for 10 minutes at 300 × g at 4 °C and then stored at -80 °C prior to use.

Purified microglia were lysed in TRIzol Reagent (Invitrogen, 15596026) and RNA was isolated using Direct-zol RNA Microprep Kits (ZYMO Research, R2062) according to manufacturer's instructions. RNA sequencing was performed by the Genome Sequencing Core of the University of Kansas using HiSeq 2000 (Illumina). The expression levels of genes were quantified by a

Kallisto program (10) by using reference transcriptome annotated from GENCODE (GRCm39, vM27). The differentially expressed genes were detected by using EdgeR program (11). Genes with a count-per-Million (CPM) of less than 1 in more than 2 samples were excluded. Pathway analysis was performed using Ingenuity Pathways Analysis (IPA) software (QIAGEN). Cellular immune response, cytokine signaling, cellular stress, and injury pathways were used in IPA analysis. The RNAseq data has been deposited in NCBI-SRA with the accession number PRJNA1019753.

Quantitative Real-time PCR

Inflammatory factors in the brains of individuals with aMCI and CU controls were assessed through real-time PCR. We extracted mRNA from frozen temporal lobe tissue obtained from CU and aMCI patients using the RNeasy Mini Kit (QIAGEN, 74104). The purified mRNA was then promptly converted into cDNA via reverse transcription using the PrimeScript RT Master Mix (TaKaRa, RR036A). Real-time PCR amplification was carried out using the SYBR Green PCR Master Mix (Applied Biosystems, 4309155) on the QuantStudio™ 5 Real-Time PCR System (Applied Biosystems). The following primer pairs were employed in this study. Interleukin 1 (*IL1*) forward: 5'-TTCGACACATGGGATAACGAGG, reverse: 5'-TTTTTGCTGTGAGTCCCGGAG; interleukin 6 (*IL6*) forward: 5'-ACTCACCTCTTCAGAACGAATTG, reverse: 5'-CCATCTTTGGAAGGTTCAGGTTG; tumor necrosis factor alpha (*TNFA*) forward: 5'-GAGGCCAAGCCCTGGTATG, reverse: 5'-CGGGCCGATTGATCTCAGC; interleukin 10 (*IL10*) forward: 5'-GACTTAAGGGTTACCTGGGTTG, reverse: 5'-TCACATGCGCCTTGATCTCTG; *GAPDH* forward: 5'-ACAACTTTGGTATCGTGGAAGG, reverse: 5'-GCCATCACGCCACAGTTTC.

Flow cytometry mitochondrial size measurement

Isolated synaptic mitochondria from CU and aMCI individuals are proceeded to flow cytometry to examine mitochondrial volume as previously described (12, 13). Mitochondria were identified with MitoTracker Green (Invitrogen, M7514). The size of mitochondria was estimated in reference to the reference beads using Flow Cytometry Sub-micron Particle Size Reference Kit (Invitrogen, F13839) in three sizes: 0.5 μm, 1 μm, and 2 μm. Mitochondria and beads were analyzed using MACSQuant® Analyzer 10 Flow Cytometer (Miltenyi Bitec). Data were analyzed with FlowJo software 10.

References:

1. Tian J, Guo L, Wang T, Jia K, Swerdlow RH, Zigman JM, et al. Liver-expressed antimicrobial peptide 2 elevation contributes to age-associated cognitive decline. *JCI Insight.* 2023;8(10).
2. Simon J. Beck LG, Aarron Phensy, Jing Tian, Lu Wang, Neha Tandon, Esha Gauba, Lin Lu, Juan M. Pascual, Sven Kroener, Heng Du. Deregulation of mitochondrial F1FO-ATP synthase via OSCP in Alzheimer's disease. *Nature communications.* 2016;7.
3. Du H, Guo L, Yan S, Sosunov AA, McKhann GM, and Yan SS. Early deficits in synaptic mitochondria in an Alzheimer's disease mouse model. *Proc Natl Acad Sci U S A.* 2010;107(43):18670-5.
4. Pelster B WC, Campos DF, Val AL. Cellular oxygen consumption, ROS production and ROS defense in two different size-classes of an Amazonian obligate air-breathing fish (Arapaima gigas). *PLos One.* 2020;15:7.
5. Du H, Guo L, Fang F, Chen D, Sosunov AA, McKhann GM, et al. Cyclophilin D deficiency attenuates mitochondrial and neuronal perturbation and ameliorates learning and memory in Alzheimer's disease. *Nat Med.* 2008;14(10):1097-105.
6. Heng Du LG, Fang Fang, Doris Chen, Alexander A Sosunov, Guy M McKhann, Yilin Yan, Chunyu Wang, Hong Zhang, Jeffery D Molkentin, Frank J Gunn-Moore, Jean Paul Vonsattel, Ottavio Arancio, John Xi Chen, Shi Du Yan. Cyclophilin D deficiency attenuates mitochondrial and neuronal perturbation and ameliorates learning and memory in Alzheimer's disease. *Nature Medicine.* 2008;14(10):1097-105.
7. Xu B HY, Liu L, Ye G, Chen L, Wang Q, Chen M, Chen Y, Long D. The Effects of Physical Running on Dendritic Spines and Amyloid-beta Pathology in 3xTg-AD Male Mice. *Aging Disease.* 2022;13(4):1293-310.
8. Kongsui R BS, Johnson SJ, Walker FR. Quantitative assessment of microglial morphology and density reveals remarkable consistency in the distribution and morphology of cells within the healthy prefrontal cortex of the rat. *Journal of Neuroinflammation.* 2014.
9. Donna M Wilcock MNG, Dave Morgan. Quantification of cerebral amyloid angiopathy and parenchymal amyloid plaques with Congo red histochemical stain. *Nature protocols.* 2006;1:1591-5.
10. Bray NL, Pimentel H, Melsted PA-O, and Pachter L. Near-optimal probabilistic RNA-seq quantification. (1546-1696 (Electronic)).

11. McCarthy DJ, Chen Y Fau - Smyth GK, and Smyth GK. Differential expression analysis of multifactor RNA-Seq experiments with respect to biological variation. (1362-4962 (Electronic)).
12. MacDonald JA, Bothun AM, Annis SN, Sheehan H, Ray S, Gao Y, et al. A nanoscale, multi-parametric flow cytometry-based platform to study mitochondrial heterogeneity and mitochondrial DNA dynamics. *Commun Biol.* 2019;2:258.
13. Schneider A, Kurz S, Manske K, Janas M, Heikenwalder M, Misgeld T, et al. Single organelle analysis to characterize mitochondrial function and crosstalk during viral infection. *Sci Rep.* 2019;9(1):8492.

Supplemental Table 1. Demographics of human samples.

Clinical Dx	Case number	Sex	Age	Post-mortem interval (PMI, Hr)	Braak score	CERAD NP
Control	39146	F	67	11.7	2	0
Control	42133	F	100	12.2	4	0
Control	42990	F	84	14	1	0
Control	45329	M	78	21	1	0
Control	46202	M	77	19.5	2	0
Control	01-31	M	81	2.8	3	0
Control	05-10	M	91	1.5	2	0
Control	05-16	M	82	3	3	0
Control	10-26	F	95	2.5	3	0
Control	10-70	M	74	3.3	1	0
Control	15-60	M	82	3.3	2	0
Control	99-02	F	70	2	2	0
Mean ± SEM		5F7M	81.8 ± 2.8	8.1 ± 2.1	2.2 ± 0.3	0 .0± 0.0
MCI	31606	F	84	10.4	3	0
MCI	40318	M	94	26.3	5	2
MCI	40449	F	79	6.5	3	2
MCI	42771	M	97	24.5	2	1
MCI	45370	M	86	13.9	2	2
MCI	02-33	M	85	2.3	4	1
MCI	05-12	F	88	2	4	0
MCI	11-08	M	96	3	3	1
MCI	11-93	M	82	2.7	2	1
MCI	12-16	F	85	12.6	4	0
MCI	12-41	M	97	2.5	4	0
Mean ± SEM		4F7M	88.5 ± 1.9	9.7 ± 2.9	3.3 ± 0.3	0.9 ± 0.3

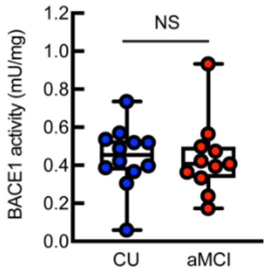

Supplemental Figure 1. BACE1 activity of CU and aMCI brain homogenates. Unpaired two-tailed *t* test. CU, *n* = 12; aMCI, *n* = 11. BACE1, β-secretase 1. NS = not significant.

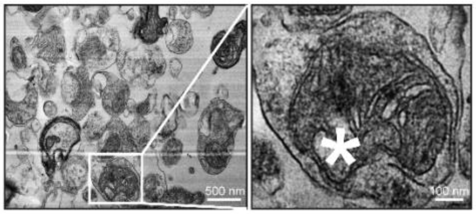

Supplemental Figure 2. Isolated human synaptosome purity. Left panel, transmission electron microscopy (TEM) image. The white box indicates the magnified view of the right panel. Scale bar = 500 nm. Right panel, ultrastructure of a synaptosome. The mitochondrion is indicated with an asterisk. Scale bar = 100 nm.

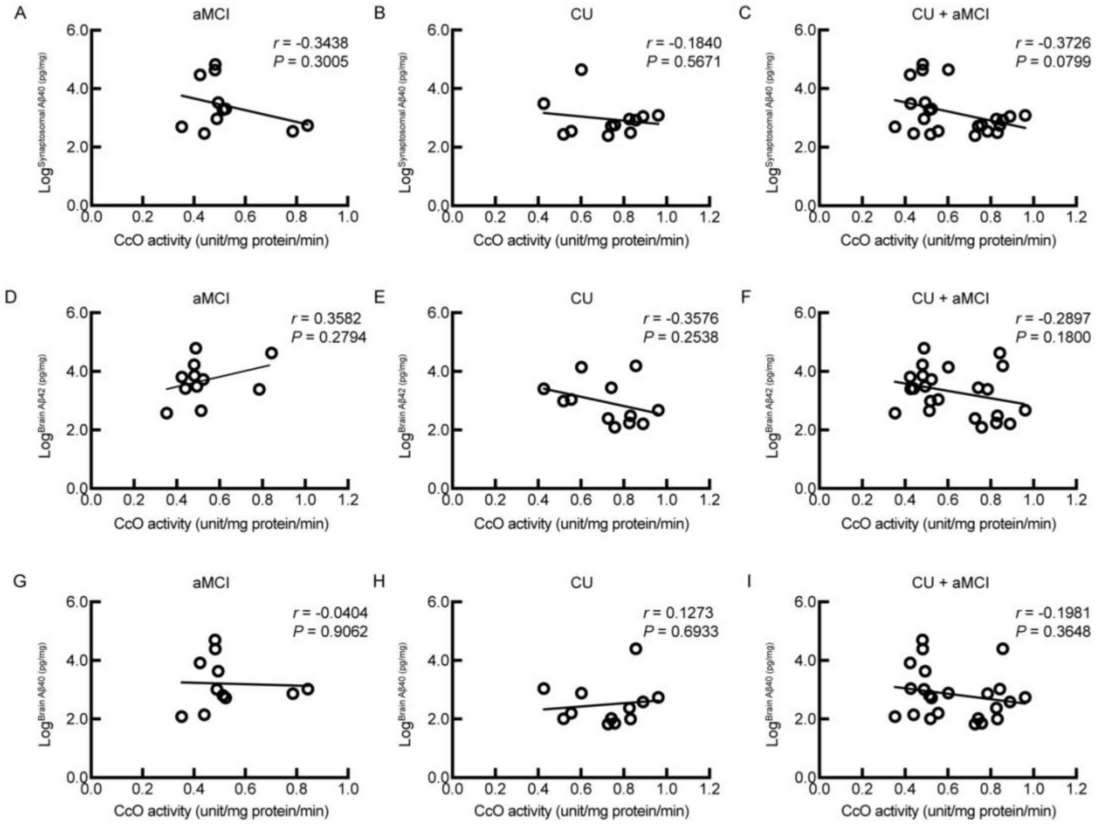

Supplemental Figure 3. Correlation of synaptosomal Aβ40, brain Aβ40, and brain Aβ42 with CcO activity in CU and aMCI subjects. (**A-C**) Correlation of synaptosomal Aβ40 with CcO activity in CU (**A**), aMCI (**B**) and combined two groups (**C**). (**D-F**) Correlation of brain Aβ42 with CcO activity in CU (**D**), aMCI (**E**), and both (**F**). (**G-I**) Correlation of brain Aβ40 with CcO activity in CU (**G**), aMCI (**H**) and both (**I**). Pearson's correlation coefficients. CU, *n* = 12; aMCI, *n* = 11.

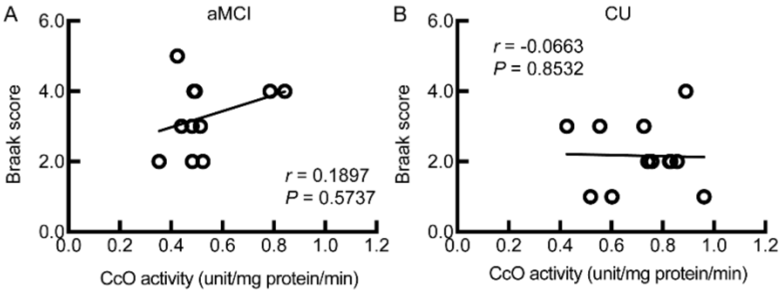

Supplemental Figure 4. Tauopathy in CU and aMCI subjects. (**A&B**) Correlation of Braak score with CcO activity in aMCI (**A**), CU (**B**). Spearman's rank correlation coefficient; CU, *n* = 12; aMCI, *n* = 11.

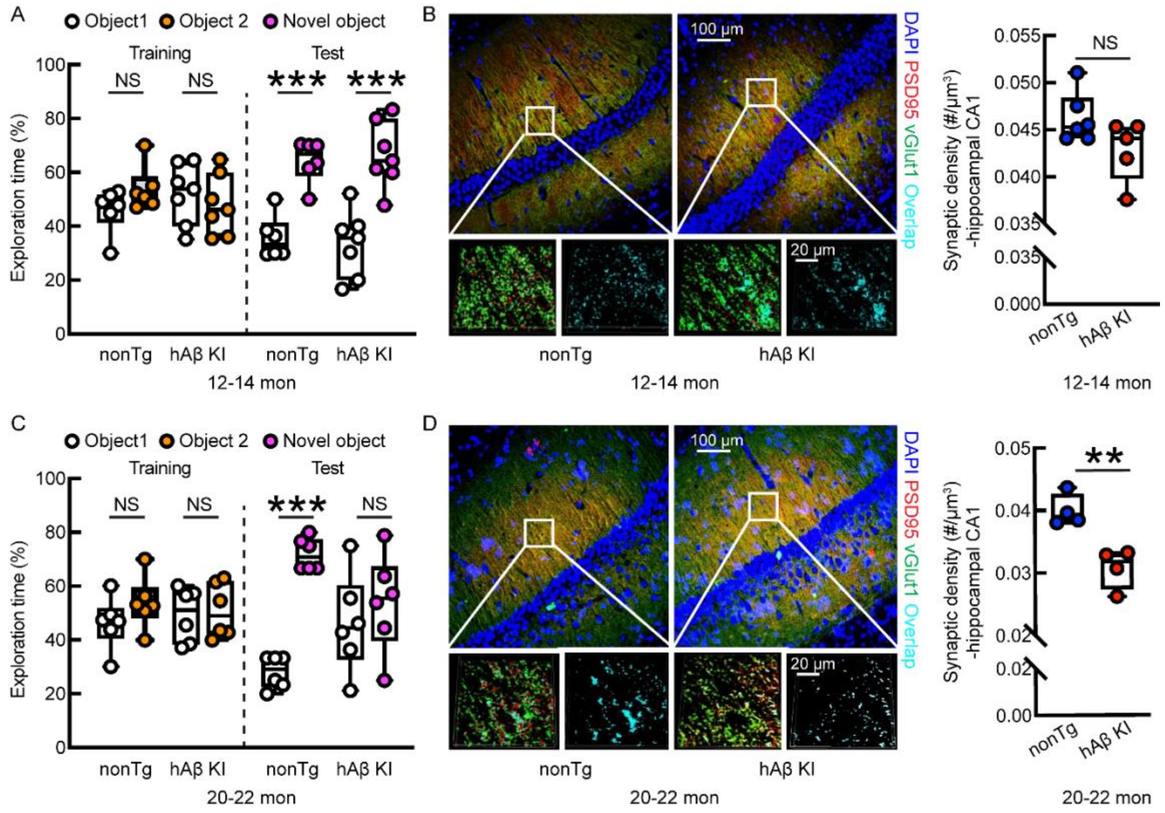

Supplemental Figure 5. Recognition memory and synaptic density in nonTg and hAβ KI mice at 12-14 months and 20-22 months. (**A&C**) Novel object test of nonTg and hAβ KI mice at 12-14 months (**A**) and 20-22 months (**C**) old. Unpaired two-tailed *t* test. nonTg, $n = 6$ for both ages; 12-14 mon hAβ KI, $n = 8$, 20-22 mon hAβ KI, $n = 7$. (**B&D**) Synaptic density in the hippocampal CA1 regions of nonTg and hAβ KI mice at 12-14 months (**B**) and 20-22 months (**D**) old. Left panel, representative images, scale bar = 100 μm (inset scale bar = 20 μm). Right panel, analysis of number of synapses per μm^3. Unpaired two-tailed *t* test. 12-14 mon, $n = 5$ each group; 20-22 mon, $n = 4$ each group. Presynaptic marker vesicular glutamate transporter 1 (vGlut1, green), postsynaptic marker postsynaptic density 95 (PSD95, red). $^{**}P < 0.01$, $^{***}P < 0.001$, NS = not significant.

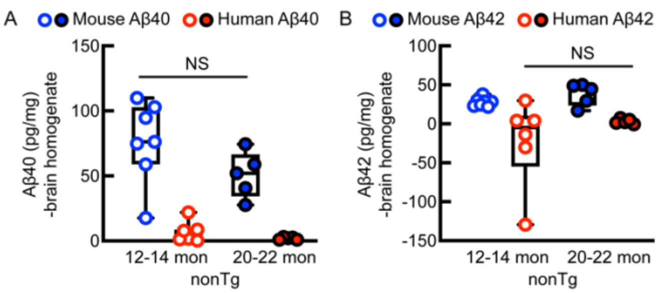

Supplemental Figure 6. Brain Aβ level in nonTg mice at 12-14 months and 20-22 months old. (**A**) Aβ40 levels in brain homogenates from nonTg mice. Unpaired two-tailed *t* test. 12-14 mon, *n* = 7 each group; 20-22 mon, *n* = 5 each group. (**B**) Aβ42 levels in brain homogenates from nonTg mice. Two-tailed *t* test with Welch's correction. 12-14 mon, mouse form, *n* = 7; human form, *n* = 6; 20-22 mon, *n* = 5 each group. NS = not significant.

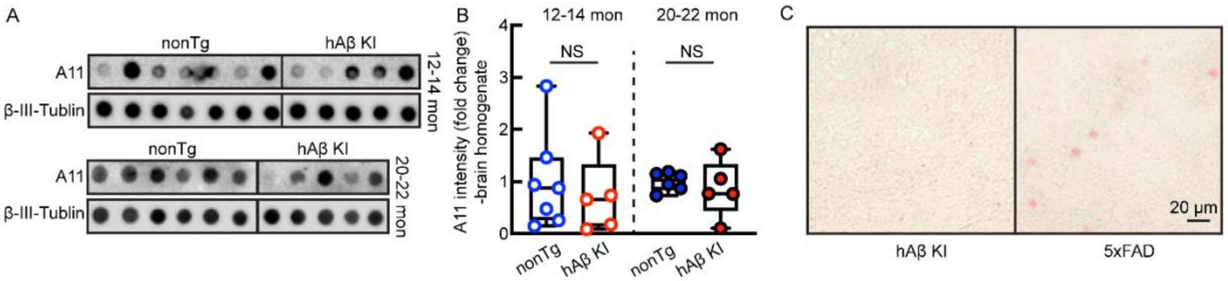

Supplemental Figure 7. Aβ oligomers and fibrils in hAβ KI mice. (**A&B**) Aβ oligomers labeled by A11 antibody. (**A**) Representative images of dot blots. 12-14 mon, unpaired two-tailed *t* test; nonTg, *n* = 7; hAβ KI, *n* = 5; 20-22 mon, two-tailed *t* test with Welch's correction; nonTg *n* = 6; hAβ KI, *n* = 5. (**B**) Densitometry analysis of immunoreactive blots. (**C**) Congo red staining of Aβ fibrils. Left panel, hAβ KI mice; right panel, 5xFAD mice. NS = not significant. Scale bar = 20 μm.

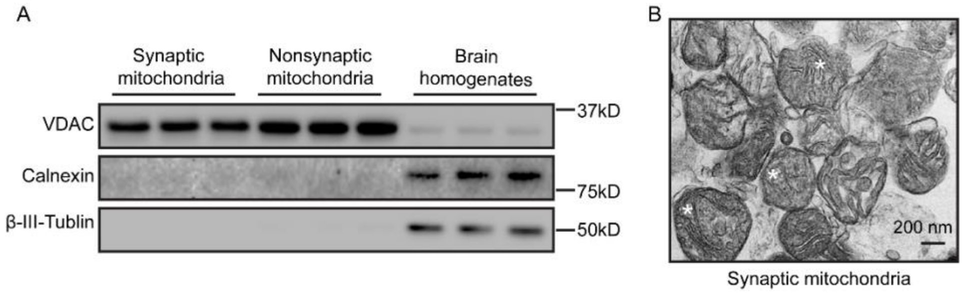

Supplemental Figure 8. Characterization of the purity of isoalted mitochondria. (**A**)Western blot images of VDAC (mitochondrial outer membrane protein), Calnexin (endoplasmic reticulum marker), and β-III-Tublin (cytosolic protein). (**B**) Electron microscopy examination of isolated synaptic mitochondria. Asterisks denote mitochondria. Scale bar = 200 nm.

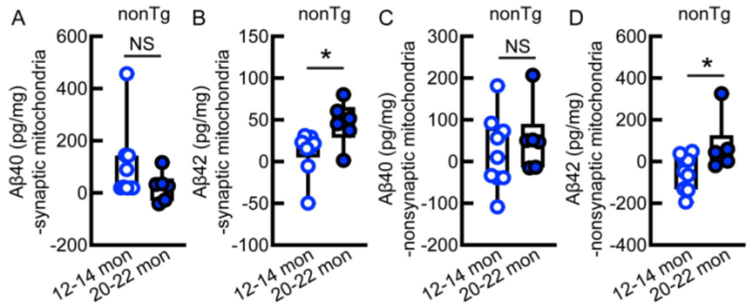

Supplemental Figure 9. Mitochondrial Aβ in nonTg mice at 12-14 months and 20-22 months. (**A&B**) Aβ40 (**A**) and Aβ42 (**B**) levels in synaptic mitochondria from nonTg mice. Two-tailed Mann-Whitney test; 12-14 mon, *n* = 9; 20-22 mon, *n* = 6. (**C&D**) Aβ40 (**C**) and Aβ42 (**D**) levels in synaptic mitochondria from nonTg mice. Unpaired two-tailed *t* test; 12-14 mon, *n* = 9; 20-22 mon, *n* = 6. **P* < 0.05, NS = not significant.

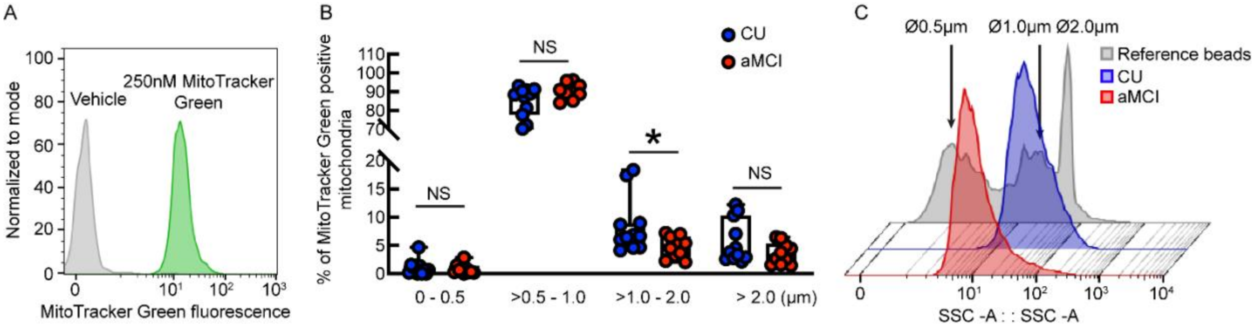

Supplemental Figure 10. Measurement of human brain mitochondrial size by flow cytometry. (A) Histogram of fluorescent intensity of vehicle and 250nM MitoTraker Green labeled mitochondria. **(B)** Percentages of MitoTracker Green-positive mitochondria with different sizes. 0 – 0.5μm and >0.5 – 1μm, unpaired two-tailed *t* test; >1.0 – 2.0μm, two tailed Mann-Whitney test; > 2.0μm, two-tailed *t* test with Welch's correction. CU, n = 11; aMCI, n = 10. **(C)** Representative histograms overlay of reference beads and mitochondria sorted by side scatter (SSC). *P < 0.05, NS = not significant.

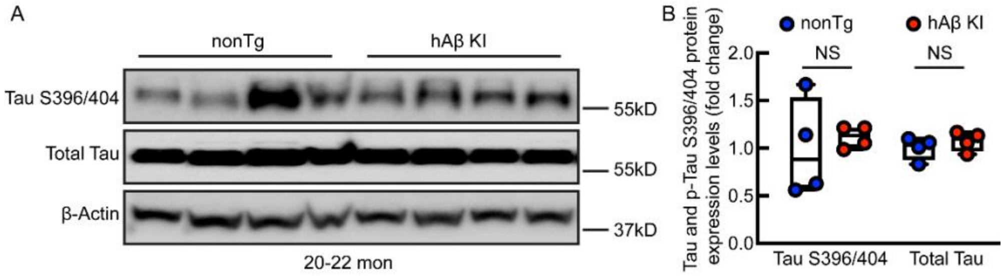

Supplemental Figure 11. Tauopathy in nonTg and hAβ KI mice at age of 20-22 months. (**A**) Full-length Western blot images. (**B**) Analysis of tau S396/404 and total tau protein expression levels of brain homogenates from nonTg and hAβ KI mice at ages of 20-22 months. P-Tau S396/404, two-tailed *t* test with Welch's correction. *n* = 4 each group. Total tau, unpaired two-tailed *t* test. *n* = 4 each group. NS = not significant.

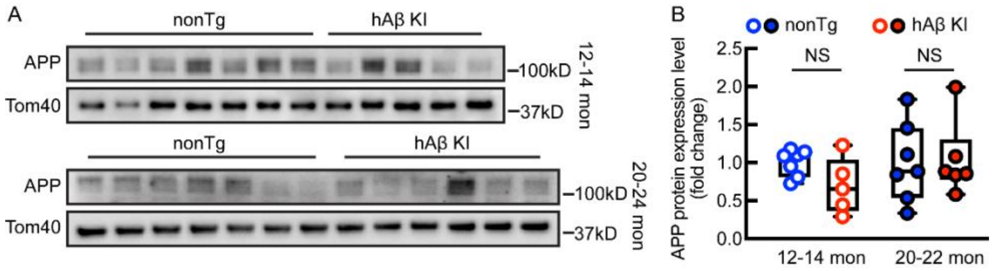

Supplemental Figure 12. Synaptic mitochondria-associated APP expression levels in nonTg and hAβ KI mice at 12-14 months and 20-22 months. (**A**) Full-length Western blot images. (**B**) Synaptic mitochondrial APP expression levels (fold change) from nonTg and hAβ KI mice at 12-14 and 20-22 months. Unpaired two-tailed *t* test. 12-14 mon, nonTg, $n = 7$; hAβ KI, $n = 5$; 20-22 mon, nonTg, $n = 7$; hAβ KI, $n = 6$. NS = not significant.

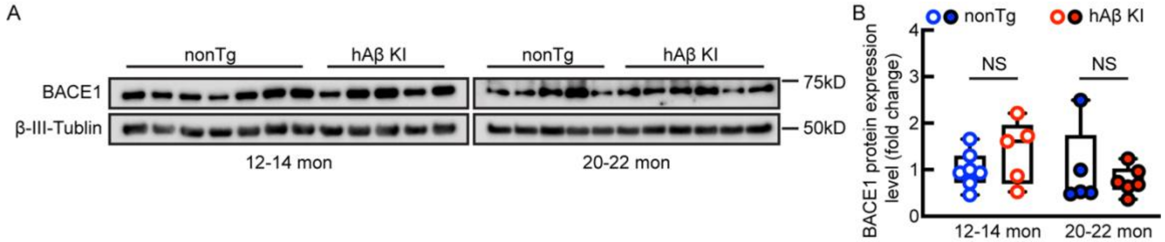

Supplemental Figure 13. BACE1 protein expression in nonTg and hAβ KI mice at 12-14 months and 20-22 months. (**A**) Full-length Western blot images. (**B**) Analysis of BACE1 protein expression levels of brain homogenates from nonTg and hAβ KI mice. 12-14 mon, unpaired two-tailed *t* test; nonTg, *n* = 7, hAβ KI, *n* = 5. 20-22 mon, two-tailed *t* test with Welch's correction; nonTg, *n* = 5, hAβ KI, *n* = 6. NS = not significant.

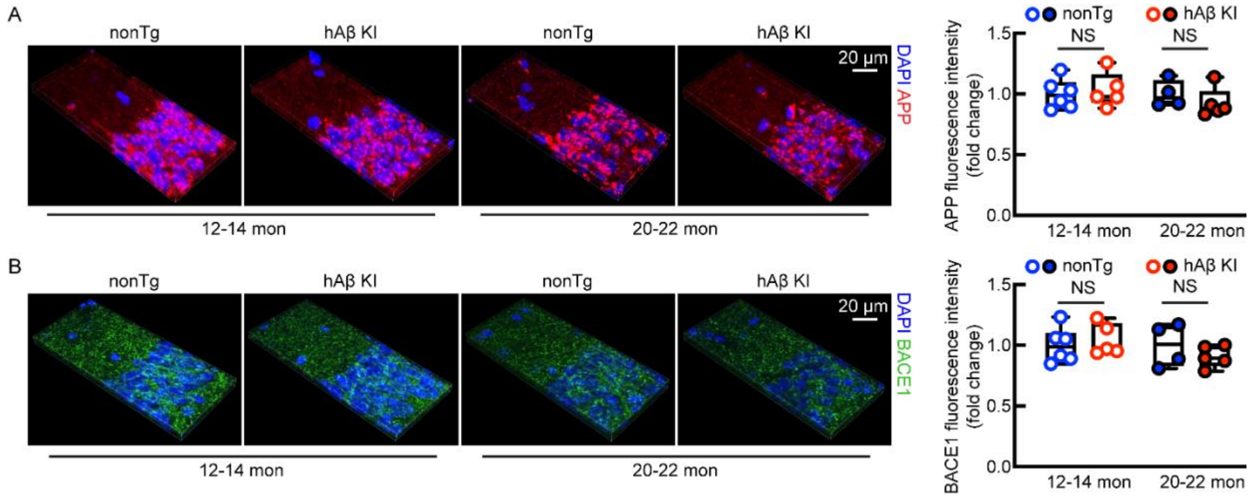

Supplemental Figure 14. Expression of BACE1 and APP of hippocampal CA1 region in nonTg and hAβ KI mice at 12-14 months and 20-22 months. (**A&B**) Left panel, representative images of hippocampal CA1 APP (**A**) and BACE1 (**B**) immunofluorescent staining, scale bar = 20 μm. Right panel, analysis of APP immunofluorescence intensity of hippocampal CA1 region. Unpaired two-tailed *t* test. 12-14 mon, *n* = 5 each group; 20-22 mon, *n* = 4 each group. NS = not significant.

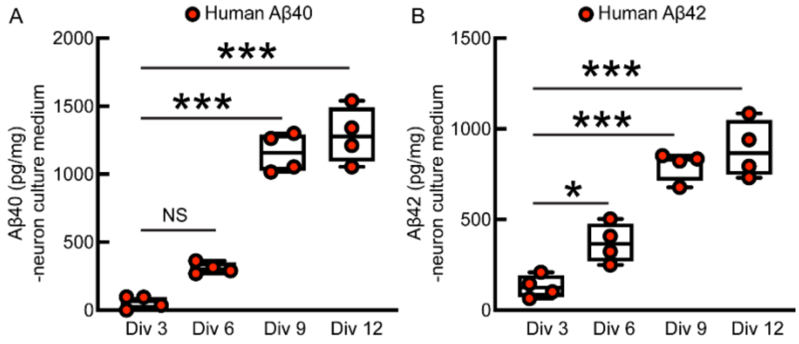

Supplemental Figure 15. Aβ levels in culture medium from hAβ KI primary cortical neuron cultures. (**A&B**) Aβ40 (**A**) and Aβ42 (**B**) levels in culture medium form primary cultured hAβ KI cortical neurons at Div 3, 6, 9 and 12. One-way ANOVA followed by Bonferroni test; $n = 4$ each group. *** $P < 0.001$.

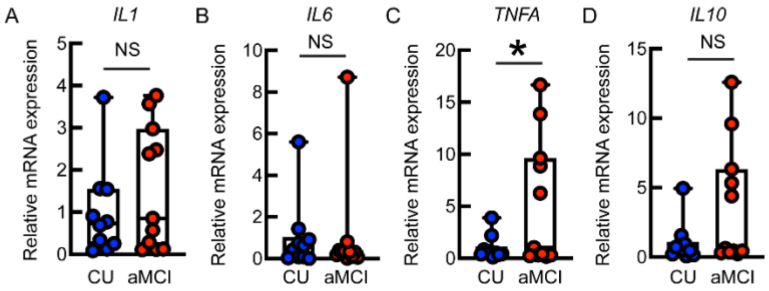

Supplemental Figure 16. Relative mRNA expression levels of inflammatory cytokines in CU and aMCI subjects. (A-D) Relative mRNA expression of *IL1* (**A**), *IL6* (**B**), *TNFA* (**C**) and *IL10* (**D**) in CU and aMCI subjects. *IL1*, unpaired two-tailed *t* test; *IL6*, two-tailed Mann-Whitney test; IL10 and TNFA, two -tailed *t* test with Welch's correction. CU, n = 10; aMCI, n = 11.
